# RBD-depleted SARS-CoV-2 spike generates protective immunity in cynomolgus macaques

**DOI:** 10.1038/s41541-025-01113-0

**Published:** 2025-03-30

**Authors:** Hélène Letscher, Delphine Guilligay, Gregory Effantin, Axelle Amen, Guidenn Sulbaran, Judith A. Burger, Laetitia Bossevot, Laura Junges, Marco Leonec, Julie Morin, Matthieu Van Tilbeurgh, Cécile Hérate, Anne-Sophie Gallouët, Francis Relouzat, Sylvie van der Werf, Mariangela Cavarelli, Nathalie Dereuddre-Bosquet, Marit J. van Gils, Rogier W. Sanders, Pascal Poignard, Roger Le Grand, Winfried Weissenhorn

**Affiliations:** 1 Université Paris-Saclay, Inserm, CEA, Center for Immunology of Viral, Auto-immune, Hematological and Bacterial diseases (IMVA-HB/IDMIT/UMR-S 1184), Fontenay-aux-Roses & Le Kremlin-Bicêtre, Paris, France; 2 Univ. Grenoble Alpes, CEA, CNRS, Institut de Biologie Structurale (IBS), Grenoble, France; 3 CHU Grenoble Alpes, Grenoble, France; 4 University of Amsterdam, Department of Medical Microbiology and Infection Prevention, Amsterdam University Medical Centers, Location AMC, Amsterdam, The Netherlands; 5 Institut Pasteur, Molecular Genetics of RNA Viruses, Department of Virology, CNRS UMR 3569, Université de Paris, Paris, France; 6 Institut Pasteur, National Reference Center for Respiratory Viruses, Paris, France; 7 Weill Medical College of Cornell University, Department of Microbiology and Immunology, New York, NY USA

**Keywords:** Biotechnology, Immunology, Diseases

## Abstract

The SARS-CoV-2 pandemic revealed the rapid evolution of circulating strains. This led to new variants carrying mostly mutations within the receptor binding domain, which is immunodominant upon immunization and infection. In order to steer the immune response away from RBD epitopes to more conserved domains, we generated S glycoprotein trimers without RBD and stabilized them by formaldehyde cross-linking. The cryoEM structure demonstrated that SΔRBD folds into the native prefusion conformation, stabilized by one specific cross-link between S2 protomers. SΔRBD was coated onto lipid vesicles, to produce synthetic virus-like particles, SΔRBD-LV, which were utilized in a heterologous prime-boost strategy. Immunization of cynomolgus macaques either three times with the mRNA Comirnaty vaccine or two times followed by SΔRBD-LV showed that the SΔRBD-LV boost induced similar antibody titers and neutralization of different variants, including omicron. Upon challenge with omicron XBB.3, both the Comirnaty only and Comirnaty/SΔRBD-LV vaccination schemes conferred similar overall protection from infection for both the Comirnaty only and Comirnaty/SΔRBD-LV vaccination schemes. However, the SΔRBD-LV boost indicated better protection against lung infection than the Comirnaty strategy alone. Together our findings indicate that SΔRBD is highly immunogenic and provides improved protection compared to a third mRNA boost indicative of superior antibody-based protection.

## Introduction

The SARS-CoV-2 spike (S) glycoprotein is the main target for inducing neutralizing antibodies. S is synthesized as a precursor polyprotein that requires proteolytic furin cleavage to generate the non-covalently linked S1-S2 heterotrimer^1^. The S1 subunit harbors the receptor-binding domain (RBD), the N-terminal domain (NTD), two subdomains (SD1 and SD2) and S2, anchoring S to the virus membrane^2^. RBD recognizes the main cellular receptor Angiotensin-converting enzyme 2 (ACE2), triggering entry by endocytosis and S2-catalyzed fusion with endosomal membranes, thereby establishing infection^3–5^. The S conformation of RBD is in a dynamic equilibrium between either all RBDs in a closed, receptor-inaccessible conformation or one or two RBDs in the “up”, receptor-accessible, conformation^4,6–9^.

First-generation licensed COVID vaccines such as Comirnaty-BNT162b2 or Spikevax-mRNA-1273 are based on the ancestral Wuhan SARS-CoV-2 virus strain and showed up to 95% protective efficacy against symptomatic COVID-19 infection with two vaccination doses^10,11^. Since then, numerous protein-based, vectored, inactivated virion-based, and mRNA LNP vaccines based on the Wuhan strain and omicron strains have been licensed or are in clinical trials with variable efficacies of protection^12^. Up to 90% of the neutralizing antibody responses generated upon SARS-CoV-2 infection and vaccination target RBD, which is highly immunodominant^13–18^.

Since the beginning of the pandemic, the ancestral Wuhan strain has mutated into a series of new variants with Alpha and Delta causing initially the majority of infections^19^ until the appearance of Omicron B.1.1.529 in late 2021^20^. Omicron evolved since then from BA.2 into several subvariants including XBB subvariants generated by recombination^21,22^. These subvariants carry more than 30 mutations within their spike, most of them within RBD, that contribute to higher transmission rates^23^ and neutralizing antibody escape^24–27^. Current subvariants XBB and BQ1.1 are most potent in escaping neutralizing antibodies (nAbs) generated by Wuhan strain S-based vaccines^27,28^. As a consequence, the monovalent SARS-CoV-2 vaccines are not effective in preventing infection^25,29–31^ nor transmission^32^, but still provide protection against severe disease^33,34^. However, the immune response can be boosted with mRNA vaccines^35,36^, and second-generation bivalent and monovalent vaccines have been developed that increase nAb titers showing cross-reactivity against Omicron^37,38^, although the generation of an effective potent immune response by new booster vaccines has been challenged^39,40^.

To circumvent the high mutation rate within RBD and focus the immune response away from RBD towards conserved epitopes of S targeted by broadly neutralizing antibodies such as the fusion peptide, the stem helix or SD1^41^, we developed S trimers from which RBD was deleted. Notably, S2-specific neutralizing antibodies exert broad in vivo protection against most pathogenic betacoronaviruses, SARS-CoV-1, SARS-CoV-2, and MERS-CoV^42–45^. Here, we used formaldehyde stabilized S coated onto lipid vesicles in a prime-boost scheme with the Comirnaty mRNA vaccine in order to evaluate whether SΔRBD lipid vesicles can efficiently boost the antibody response and provide protection from infection in a non-human primate model.

## Results

### Structure of SΔRBD and lipid vesicle coating

**Fig. 1:**
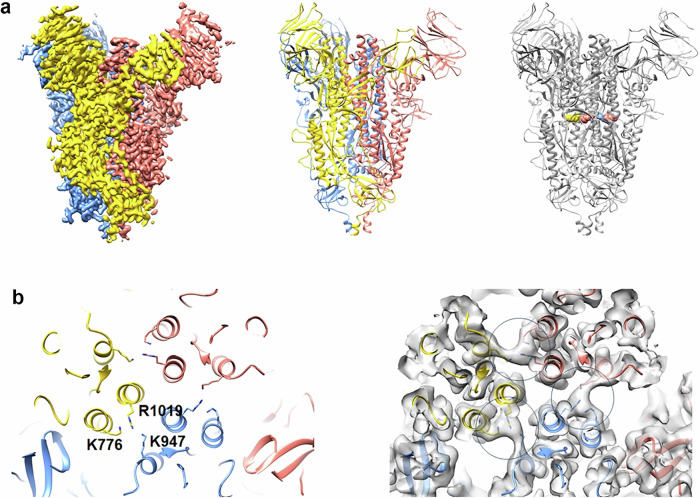
Structural characterization of cross-linked SARS-CoV2 SΔRBD. **a** Right panel, cryo-EM coulomb potential map of SΔRBD; each protomer is colored differently. Middle panel, molecular model of SΔRBD shown as ribbon. Right panel, one major cross-linking site was identified that covalently links the S2 subunits from different protomers. **b** Close-up of the cross-linking sites between S2 subunits (left panel). Continuous density between the central helix R827 as well as S2 K584 to S2 HR1K755 suggested two alternative cross-links between protomers with equal occupancy (right panel).

The S glycoprotein construct ‘6 P’^46^ without RBD (SΔRBD) was expressed in mammalian cells and purified by Ni^2+^-affinity and size exclusion chromatography (SEC) (Supplementary Fig. 1A). Because SΔRBD showed a Tm of 44.5 °C in comparison to 63.4 °C for S ‘6 P’, SΔRBD was stabilized by chemical cross-linking with 4% formaldehyde (FA). This produced a high molecular weight species as determined by SDS-PAGE (Supplementary Fig. 1A). FA-SΔRBD revealed an increased Tm of 56.3 °C, indicating successful stabilization by cross-linking. Negative staining electron microscopy analyses demonstrated the native prefusion conformation of FA-SΔRBD (Fig. S1B), and its structure was subsequently solved at 3.2 Å resolution by cryo-electron microscopy confirming the prefusion S conformation (Fig. 1a, Supplementary Fig. 2, and Supplementary Table 1). The structure revealed one major cross-linking site between residues R1019 of the central S2 helix and/or S2 K776 and S2 HR1 K947 from neighboring protomers (Fig. 1a, b), which stabilized S in the prefusion conformation. Furthermore, the SΔRBD structure is identical to full-length S revealing an r.m.s.d of 0.8 Å between SΔRBD and S (pdb 7q1z), indicating that all non-RBD epitopes are conserved. FA SΔRBD was incubated with liposomes (Phosphatidylcholine 60%, cholesterol 36%, DGS-NTA 4%), and efficiently captured via its C-terminal His-tag. Free, unbound FA-SΔRBD was removed from the FA-SΔRBD proteoliposomes by sucrose gradient centrifugation (Supplementary Fig. 1C), and decoration of the liposomes with FA-SΔRBD (SΔRBD-LV) was confirmed by negative staining electron microscopy (Supplementary Fig. 1D).

### Antibody responses in cynomolgus macaques

**Fig. 2:**
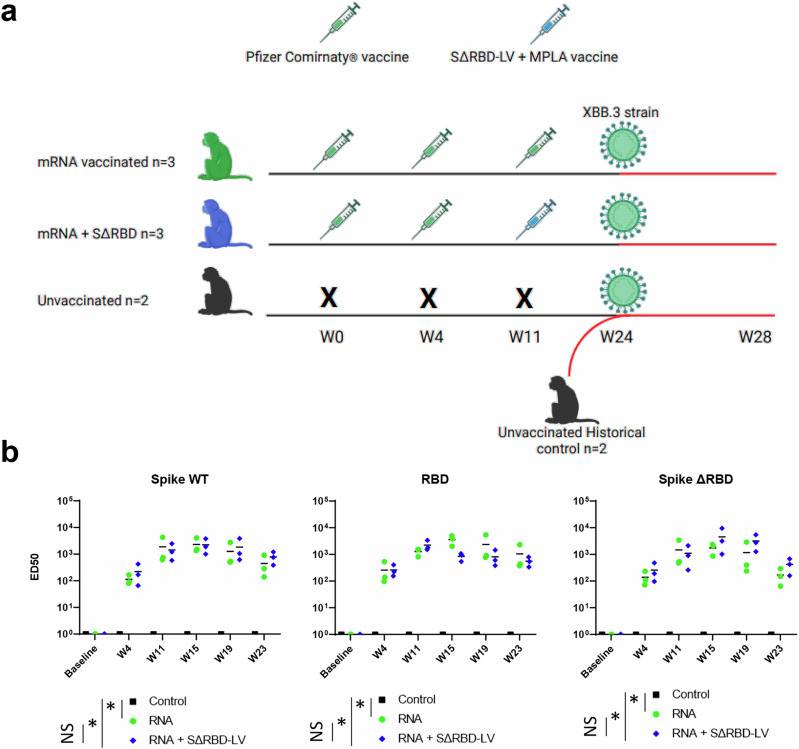
Antibody responses induced by vaccination of cynomolgus macaques. **a** Scheme of vaccination, challenge and sampling. Syringes indicate the time points of vaccination, with the green syringe representing the Comirnaty vaccine and the blue one SΔRBD-LVs. The control group (unvaccinated) included two control animals from the start. The data from two “historical” controls were added after challenge (Supplementary Table 2). The virus particle represents the time point of the challenge. Symbols of identifying individual macaques are used in all figures. **b** Serum antibody titers specific for soluble WT S glycoprotein, SΔRBD and RBD in ELISA were determined during the study at weeks 0, 4, 11 and 15. BL = baseline. Differences between groups were compared using the Mann–Whitney non-parametric rank test (control group, unvaccinated, black square).

To assess the efficacy of SΔRBD as a booster candidate, we immunized one group (*n* = 3) of cynomolgus macaques three times (at weeks 0, 4, 11) with the Pfizer/BioNTech Comirnaty vaccine (Comirnaty group). The second group (*n* = 3) received two doses of Comirnaty followed by a SΔRBD-LV boost (SΔRBD group) adjuvanted with monophospholipid A (MPLA) liposomes by the intramuscular route, and the third group (*n* = 2) was non-vaccinated receiving only PBS at each injection (Fig. 2a and Supplementary Table 2). No weight loss was observed, and no abnormality was recorded in the blood cellular formula during the 8 weeks following the immunization of the animals (Supplementary Fig. 3).

The sera of the immunized macaques were analyzed for binding to native the S glycoprotein (S), to the RBD and to the SΔRBD. This analysis showed similar S-specific Ab titers for all vaccinated animals. The ED50 titers for S, RBD and SΔRBD are similar at week 4 and week 11 with slight variations between animals, since both groups were immunized twice with Comirnaty. In general, ED50 titers increased by a factor of ~2000 from week 4 to week 11 after the second immunization (Fig. 2b). After the third immunization, ED50 S titers were at approximately the same level in both groups on week 15, similar to the week 11 titers. As expected, on week 15, ED50 RBD titers increased by a factor of 4 in the Comirnaty group, but diminished by a factor of 2 in the SΔRBD group, while the SΔRBD titers increased 4-fold in the SΔRBD-LV group while remaining stable in the Comirnaty group (Fig. 2b). Furthermore, Ab titers decreased in both groups on week 19 and 23, respectively 8 and 12 weeks after the third immunization, as observed in many studies before. However, the decrease in Ab titer against S and SΔRBD was less pronounced in the SΔRBD group compared to the decrease in the Comirnaty group revealing an approximately 2 to 3-fold difference in S and SΔRBD S titers between the groups (Fig. 2b). This suggests that SΔRBD immunization redirects the immune response to non-RBD epitopes resulting in overall similar S-specific Ab titers.

**Fig. 3:**
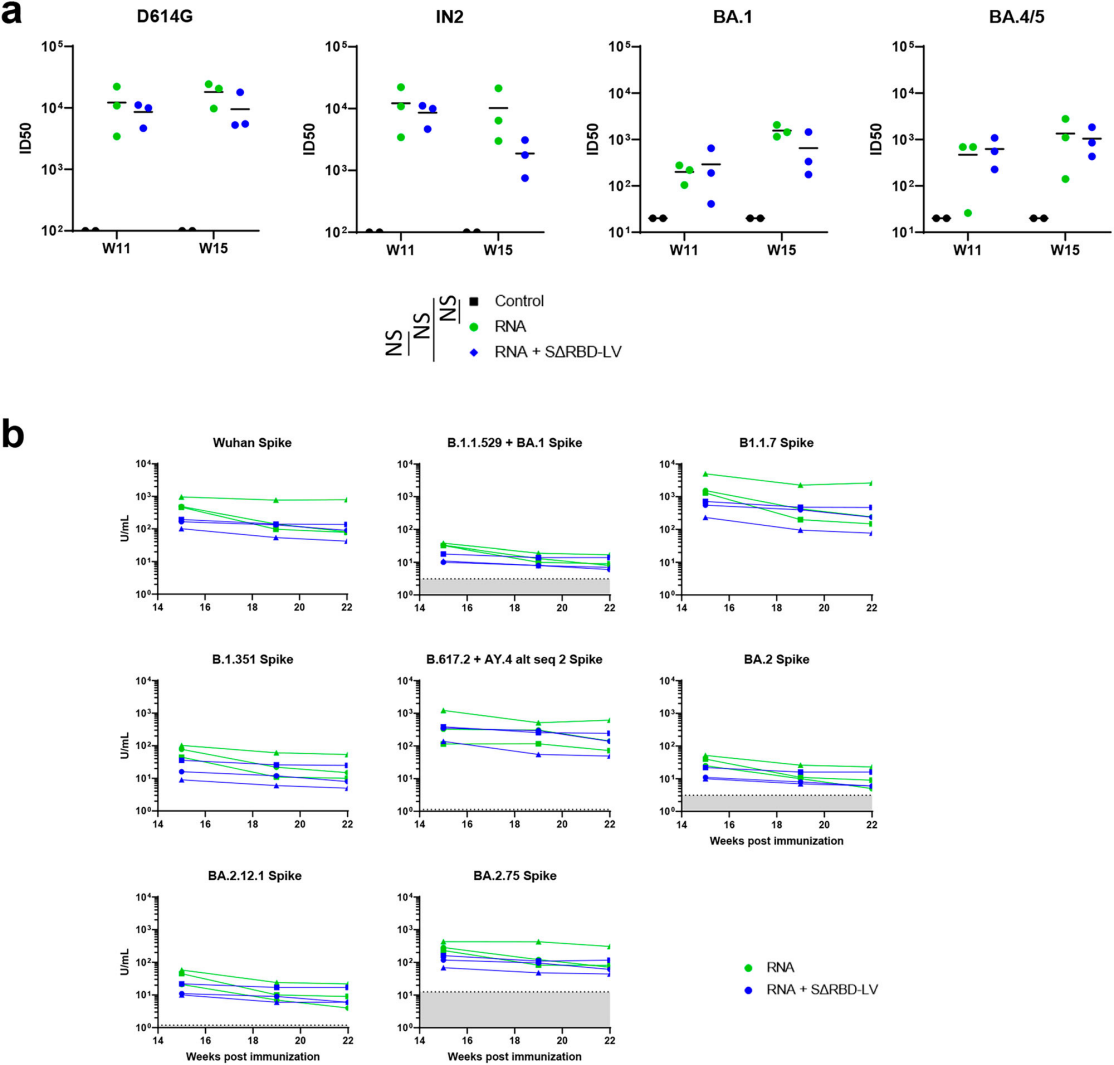
Serum neutralization of SARS-CoV-2 pseudovirus upon SΔRBD-LV vaccination. **a** Neutralization of various SARS-CoV-2 strains is shown for sera collected at weeks 11 and 15. Bars indicate mean titers for the three animals. Neutralization is expressed as inhibitory dilution at which 50% neutralization is achieved. Differences between groups were compared using the Mann-Whitney non-parametric rank test. The data presented are from technical duplicates. **b** ELISA-assessed neutralizing titer of spike-specific Ab against several SARS-CoV-2 variants on weeks 15, 19 and 22. Each curve represents an animal. Differences between groups were compared using the Mann–Whitney non-parametric rank test. Gray shaded area: lower limit of quantification (LLOQ). When data were lower than the LLOQ, they were adjusted to the LLOQ. The units of the *y*-axis (U/mL) are arbitrary units per mL.

Significant serum neutralization titers were elicited in both groups after the second immunization, and these were further boosted by the third immunization. On week 15, the pseudovirus neutralization titers against the D614G mutant showed mean ID50 titers of 18188 for the Comirnaty group and 9549 for the SΔRBD group after the third immunization. Mean ID50 titers against IN2 (Delta) were 10231 and 1873 for the Comirnaty and SΔRBD groups, respectively. Against Omicron variants BA1, the mean ID50 titers were 1550 and 655, and for BA4/5 they were 1340 and 1044 for both groups, respectively (Fig. 3a). Viral neutralization was also assessed in RBD-specific antibody-depleted sera from week 15, yet, no statistically relevant difference was observed between the two groups (Supplementary Fig. 4). Neutralization titers were also assessed using the ACE-2 pseudo neutralization assay, which revealed potent neutralization against the Wuhan and B1.1.7 (Alpha) strains for both groups with sera from the Comirnaty group sera exhibiting higher potency. Neutralization against Omicron B.1.1.529/BA.1, BA.2, and BA.2.12.1 were very weak in both groups, while neutralizing titers against B.617.2 (Delta) and BA.2.75 (Omicron) were comparable to those against the ancestral Wuhan strain (Fig. 3b). We conclude that one boost with the SΔRBD-LVs induces comparable neutralization profiles, albeit slightly possibly reduced for some variants, suggesting that SΔRBD-LVs activate antibodies targeting neutralization epitopes outside the RBD.

### Vaccine-induced T-cell response

**Fig. 4:**
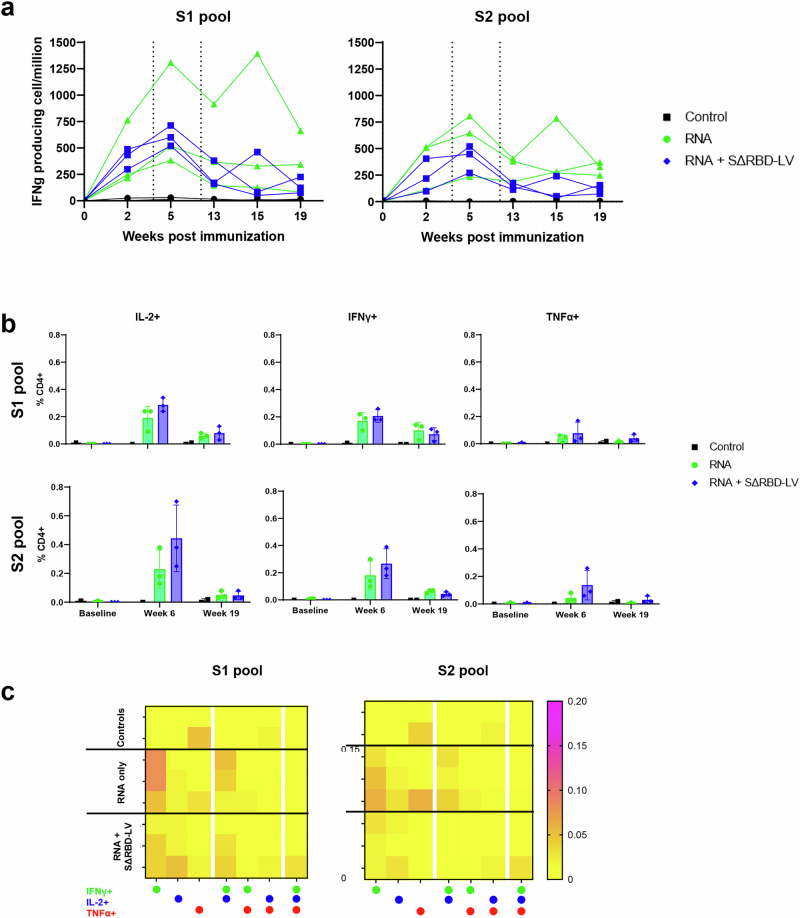
SΔRBD-LV immunization elicits a potent cellular response. **a** Spot-forming units detected by IFNγ ELISPOT in peripheral blood mononuclear cells drawn on weeks 0, 2, 5, 13, 15, and 19 after vaccination and stimulated with peptides spanning SARS-CoV-2 spike protein for 24 h. Data are represented as mean ± SD. Differences between matched groups were compared using the Mann–Whitney non-parametric rank test. The data presented are from technical duplicates. Vertical dashed lines represent time points of immunization. **b** Flow cytometry characterization of T cell response from baseline, weeks 6 and 19 after immunization. Frequency of IFNγ+, IL-2+, and TFNα+ antigen-specific CD4 + T cells in the total CD4 + T cell population. PBMCs were stimulated overnight with SARS-CoV-2 S overlapping peptide pools. Each dot represents a single animal. Data are represented as mean ± SD, means are indicated by bars. Differences between matched groups were compared using the Mann–Whitney non-parametric rank test. **c** Heat map of Th-1 polyfunctionality of T-cells stimulated with the S1 or S2 pools was assessed 8 weeks after the third immunization. The left columns correspond to monofunctional CD4+T cells, respectively IFN-γ+, IL-2+, and TNFα+ single producing cells amongst CD4+T cells, center columns bifunctional CD4+T cells, respectively IFN-γ+/IL-2+, IFN-γ+/TNFα+ and IL-2+/TNFα+. The right column is a complete polyfunctional IFN-γ+/IL-2+/TNFα+. The percentage of cytokine-positive cells increases as the color changes from yellow (0%) to purple (0.2%).

T-cell responses were observed in all vaccinated macaques following ex vivo stimulation of PBMCs with S-peptide pools, with no significant difference between the Comirnaty or SΔRBD-LV vaccinated groups (Fig. 4a). The response was highest on week 5, one week after the second immunization for each vaccinated group with a peak of 736 ( ± 499) IFN-γ producing cells per million in the Comirnaty group and 611 ( ± 97) for the SΔRBD group. IFN-γ producing cells subsequently decreased, followed by an increase on week 15, the final Comirnaty or SΔRBD-LV boosts, which revealed a secondary peak in one animal of each group in response against both S1 and S2 pools. Overall, animals in both groups tend to have a lower T-cell response after the third immunization compared to the first or second immunization. Vaccine-specific T cell subtypes were also investigated using intracellular staining of expressed cytokines (Supplementary Fig. 5). After three immunization doses, specific CD4 + T cells producing mostly either IFNγ or co-producing IFNγ and IL-2 were observed against both the S1 and S2 peptide pools in all vaccinated animals with no significant difference between the groups (Fig. 4b, c). Furthermore, none of the groups had detectable anti-S CD8^+^ T cells (Supplementary Fig. 6) nor major T-cell sublineage proportion modification (Supplementary Fig. 7).

### Challenge with Omicron variant

**Fig. 5:**
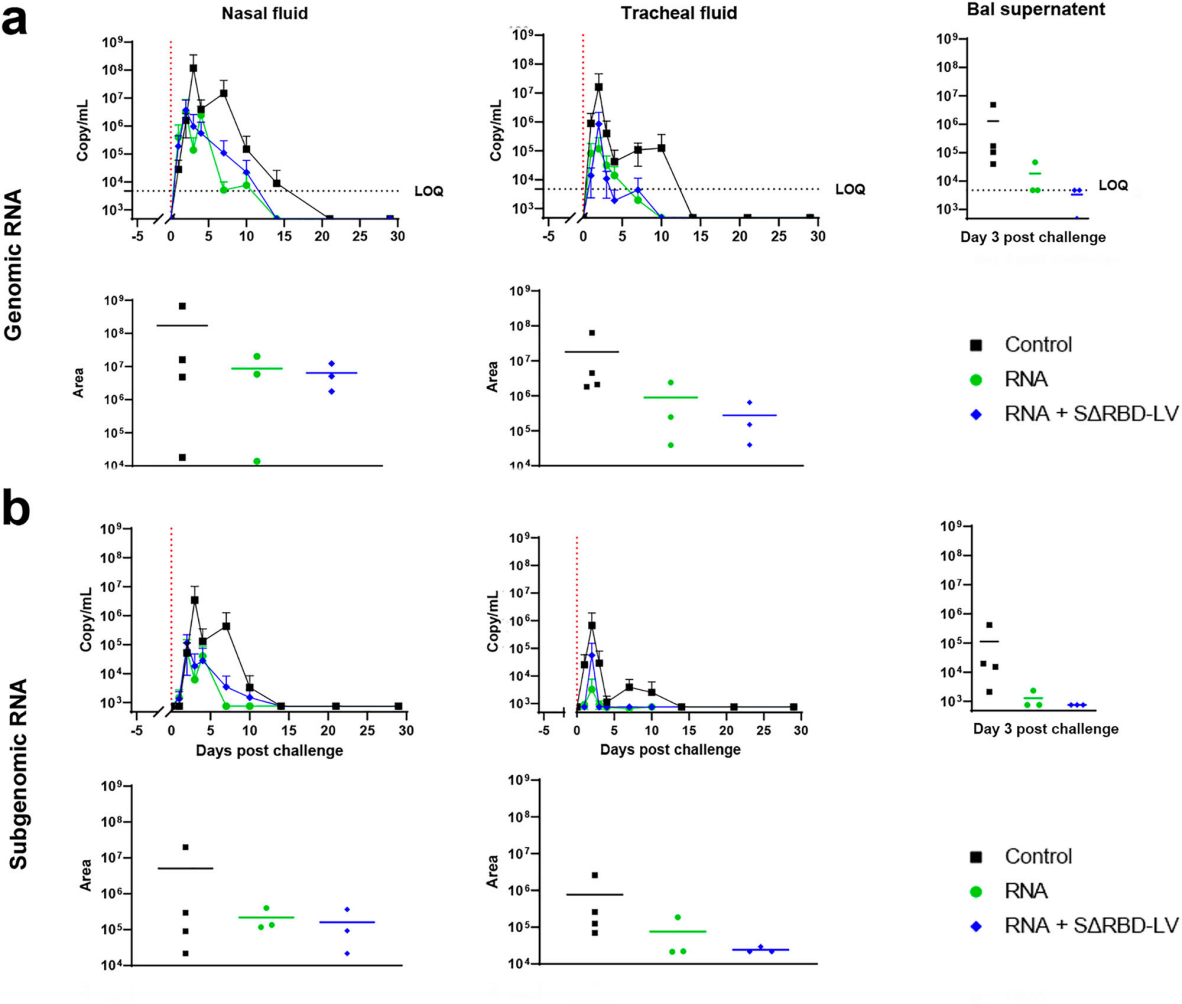
Protection from SARS-CoV-2 challenge in vaccinated cynomolgus macaques. Nasopharyngeal, tracheal, and broncho-alveolar SARS-CoV-2 viral loads. Genomic (**a**) and subgenomic (sg) (**b**) RNA viral loads were measured in fluids. In each case, the top row indicates viral burden measures by PCR while the lower row indicates the AUC calculated for each condition in tracheal swabs (left), nasal swabs (middle) and broncho-alveolar fluids (right) of control (black), RNA vaccinated (green) and RNA vaccinated, SΔRBD boosted (blue) macaques after challenge. Bars indicate mean viral loads. Vertical dotted lines indicate the day of challenge. Data are represented as mean and ±SD. When data were between the LOD and the LOQ, they were adjusted to the LOQ. Data presented are means from technical duplicates. The dotted line represents the lower limit of quantification (LOQ) when applicable.

On week 24, 13 weeks after the third immunization with the Comirnaty vaccine, the SΔRBD and control (*n* = 4) groups were challenged with a total dose of 1 × 10^5^ plaque-forming units (pfu) of the heterologous XBB.3 strain, a BA.2.75 omicron variant, by combining intranasal (0.25 mL into each nostril) and intratracheal (4.5 mL) routes. Viral load in the control group peaked in the trachea 2 days post-challenge (dpe) with a mean peak viral load of 1.61.10^7^ (±3.015.10^7^) copies per mL and an area under the curve (AUC) of 1.8.10^7^ (±3.05.10^7^). Complete clearance was observed at 14 dpe. Similar profiles were observed in both vaccinated groups. The Comirnaty group showed a peak viral burden of 1.17 × 10^5^ ( ± 1.64.10^5^) copies per mL at 2 dpe and an AUC of 2.79.10^5^ ( ± 3.23.10^5^). The viral load of the SΔRBD group peaked at 2 dpe with 8.43 × 10^5^ ( ± 1.28.10^6^) copies per mL at 2 dpe and an AUC of 8.97.10^5^ ( ± 1.31.10^6^). The SARS-CoV-2 virus remained detectable until day 14 post-challenge in both vaccinated groups (Fig. 5a). Genomic RNA was also detected in nasal fluids, with both vaccinated groups exhibiting similar profiles. The control group peaked at 3 dpe with 1.17 × 10^8^ ( ± 2.34.10^8^) copies per mL with an AUC of 1.72.10^8^ ( ± 3.30.10^8^) and complete clearance at 21 dpe. Both Comirnaty and SΔRBD groups peaked at 2 dpe with a mean of 3.25 × 10^6^ ( ± 5.59.10^6^) copies per mL and 3.71.10^6^ ( ± 4.73.10^6^) copies per mL respectively, and an AUC of 8.72.10^6^ ( ± 1.04.10^7^) and 6.38.10^6^ ( ± 5.36.10^6^) respectively. Complete viral clearance was observed in both groups at 14 dpe.

In the bronchoalveolar lavage (BAL), the four control animals showed significant viral loads at 3 dpe with a mean of 1.28 × 10^6^ ( ± 2.35.10^6^) copies/mL. All animals of the Comirnaty group exhibited detectable genomic RNA in the BAL even though only one had an above limit of quantification (LOQ) viral burden. The three RNA + SΔRBD-LV vaccinated animals had a below LOQ viral load with 1 at the limit of detection range, and 1 below detection range (Fig. 5a).

Viral subgenomic RNA (sgRNA), which estimates the number of infected and productively infected cells, confirmed a similar behavior in both vaccinated groups. The control group showed peak copy numbers in tracheal fluids at 2 dpe with mean of 6.71×10^5^ ( ± 1.22.10^6^) copies/mL and an AUC of 7.65.10^5^ ( ± 1.23.10^6^), whereas in the vaccinated groups, a viral load mean peak of 3.27 × 10^3^ ( ± 4.36.10^3^) copies/mL for the Comirnaty group and 5.53×10^4^ ( ± 9.45.10^4^) copies/mL for the SΔRBD group. Both vaccinated groups had consistent AUC with 2.42.10^4^ ( ± 4.40.10^3^) for the Comirnaty group and 7.63.10^4^ ( ± 9.45.10^4^) copies per mL for the SΔRBD group were observed. Similar subgenomic RNA copy numbers were detected in nasal fluids, with mean peak viral load of 3.42×10^6^ ( ± 6.84.10^6^) copies/mL and a AUC of 5.08.10^6^ ( ± 9.88.10^6^) in the control group, 5.49 × 10^4^ ( ± 9.37.10^4^) copies/mL with an AUC of 1.60.10^5^ ( ± 1.81.10^5^) in the Comirnaty and 1.13 ×10^5^ ( ± 1.04.10^5^) with an AUC of 2.17.10^5^ ( ± 1.59.10^5^) in the SΔRBD groups. BAL revealed 1.12 × 10^5^ ( ± 2.00.10^5^) copies per mL in the control group, 1.2×10^3^ ( ± 909) copies per mL in the Comirnaty group, and sgRNA below the detection limit in the SΔRBD group (Fig. 5b). Values of of gRNA and sgRNA of individual animals are shown in Supplementary Fig. 8. In summary, the detection of genomic and subgenomic RNA indicates that vaccination reduces viral load by 1 to 2 logs in both vaccinated groups, although the difference did not reach statistical significance due to the low number of animals, notable variations between individuals and the non-parametric nature of the statistical tests.

**Fig. 6:**
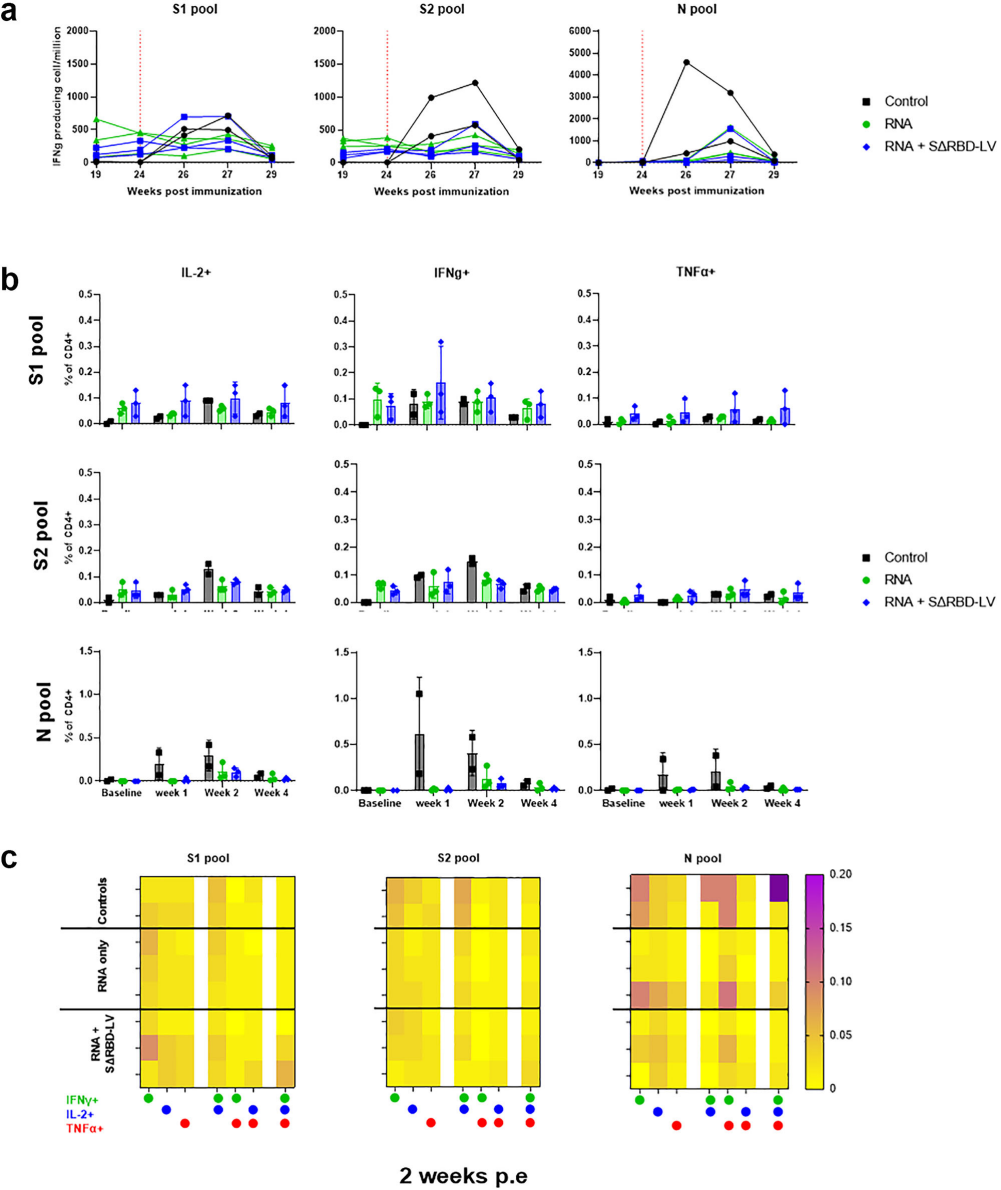
Cellular response in SARS-CoV-2 challenged macaques. **a** Spot-forming unit detected by IFNγ ELISPOT in peripheral blood mononuclear cells drawn before challenge and at weeks 1, 2, and 4 post challenge, corresponding to weeks 23, 25, 26, and 28 after the first immunization) post exposure with XBB.3 variant. Cells were stimulated with peptides spanning SARS-CoV-2 spike protein for 24 h. Differences between groups were compared using the Kruskal–Wallis non-parametric rank test of the area under curve tests, but none were significant. Data presented are from technical duplicates; the red dotted line indicates the date of the challenge. **b** Flow cytometry characterization of T cell response from baseline, week 1, week 2 and 4 after challenge. Frequency of IFNγ+, IL-2+, and TFNα+ antigen-specific CD4 + T cells in the total CD4 + T cell population. PBMCs were stimulated overnight with SARS-CoV-2 S overlapping peptide pools. Bars indicate mean values. Each dot represents a single animal. Data are represented as mean values plus ± SD. Differences between matched groups were compared using the Kruskal–Wallis rank test. **c** Heat map of Th-1 poly-functionality of T-cells stimulated with the S1 or S2 pools was assessed 8 weeks after the third immunization. Left columns, mono-functionality, respectively IFN-γ+, IL-2+, and TNFα+ single producing cells amongst CD4 + T cells, center columns bi-functionality, respectively IFN-γ+/IL-2+, IFN-γ+/TNFα+ and IL-2+/TNFα+. The right columns show complete polyfunction IFN-γ+/IL-2+/TNFα+. The results are presented as a heat map. The percentage of cytokine-positive cells increases as the color changes from yellow (0%) to purple (0.2%). One value exceeded this range of color (0.26%) and was allowed to be marked as darker purple.

Specific IFN-γ producing cells in the blood were analyzed after a challenge with the Omicron variant. No significant difference was observed between the vaccinated groups, both against S1- and S2- stimulation at day 14 post challenge, as previously observed^47^ in an anamnestic response (Fig. 6a). Furthermore, the vaccinated animals from both groups demonstrated a limited response to the N peptide pool stimulation. In contrast, the control animals exhibited a de novo anti-S1, -S2, and -N Th1 CD4+ response 1 to 2 weeks post challenge, (Fig. 6b). This response was mostly IFNγ/IL-2 dependent after stimulation with S1 and S2 pools, with no difference between the two vaccinated groups. Conversely, the de novo N-specific response was IFNγ/TNFα oriented in every group, with one control animal exhibiting a strong trifunctional T cell response (Fig. 6c). No CD8 T cell activation was observed after the challenge, regardless of the group (Supplementary Fig. 6).

**Fig. 7:**
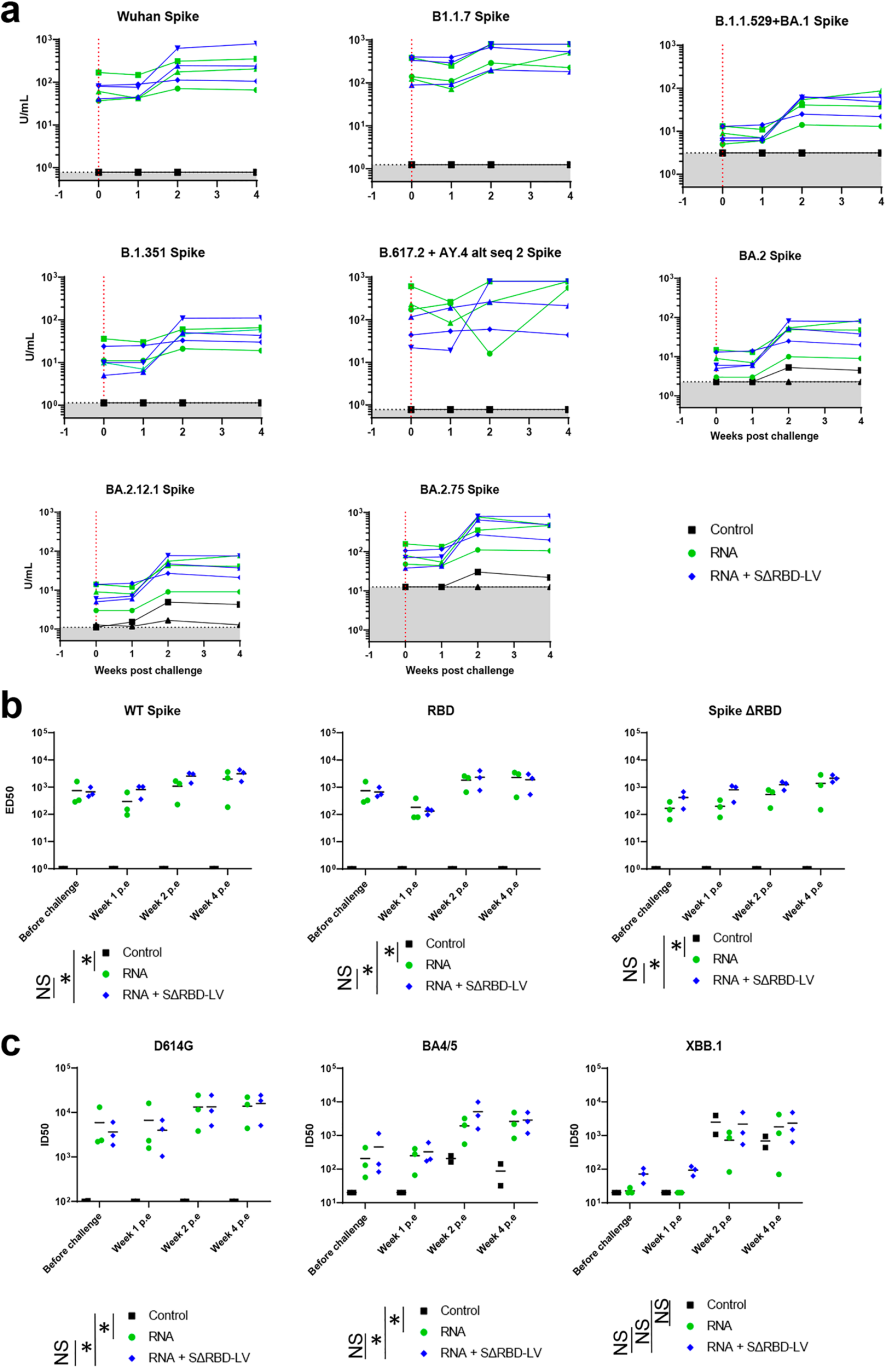
Neutralizing antibody response in challenged macaques. **a** Neutralizing Ab titer evolution of spike-specific Ab against several SARS-CoV-2 variants before the challenge and 1, 2, and 4 weeks after the challenge was assessed using an ACE-2 competition assay. Each curve represents an animal. Differences between groups were compared using the Kruskal–Wallis signed-rank test. The red dotted line indicates the challenge date, and the gray shaded area is the lower limit of quantification (LLOQ). When data were lower than the LLOQ, they were adjusted to the LLOQ. The dashed vertical line indicates the date of the challenge. The units of the *y*-axis (U/mL) are arbitrary units per mL. **b** Serum antibody titers specific for soluble WT S glycoprotein, SΔRBD, and for RBD in ELISA were determined during the study before challenge (week 23, i.e., 1 week before exposure) and 1, 2, and 4 weeks after challenge. Mean Ab titers of two experiments of individual animals are shown. Differences between matched groups were compared using the Mann-Whitney non-parametric rank test. p.e. post exposure. **c** Various SARS-CoV-2 strain virus neutralization are shown for sera collected at weeks 11 and 15. Bars indicate the mean titers of the three animals. Neutralization is expressed as inhibitory dilution at which 50% neutralization is achieved. Differences between groups were compared using the Mann–Whitney non-parametric rank test. The data presented are from technical duplicates.

An electrochemiluminescence *(*ECL)-based ACE2 competition assay employing different S variants was used to assess pseudoneutralizing potency of the antibody pool. This assay included Wuhan, BA.2.75 (omicron, subvariant of the challenge virus XBB.3), B1.1.7 (alpha), B.617.2 +Ay alt seq 2 spikes (delta), B.1.1.529 + BA1 (omicron), BA.2 (omicron), B.1.351 (omicron) (BA.2.12.1 (omicron) and BA.5 (omicron) variants. A mean 10-fold boost effect against most variants was observed two weeks after the challenge (week 26), with a more pronounced effect against more recent variants, except for B.617.2 and AY.4 (Fig. 7a). In addition, ED50 antibody titers against S, SΔRBD, and RBD were measured to represent antibody binding against those targets. The response in both vaccinated groups remained stable up to 4 weeks post-challenge, while no response was observed in the control group. No significant difference could be observed between the two vaccinated groups in terms of antibodies targeting either RBD or SΔRBD (Fig. 7b). Neutralization assays showed no significant modification of the neutralizing antibodies against D614G but a ~ 10 to 15-fold increase against BA4/5 at 2 weeks pe, and a ~ 100-fold increase against XBB.1 in each group compared to neutralization before challenge (week 23), which is expected, considering XBB.1 is the virus used for challenge. Interestingly, unlike the Comirnaty vaccine group, the SΔRBD group presented an initial XBB.1 neutralizing response before the challenge. However, by two weeks pe, all three groups showed similar neutralizing responses. The neutralization responses against BA4/5 and XBB.1 were maintained in both vaccinated groups, whereas it tended to decline in the control group (Fig. 7c). Thus, the general polyvalence of the neutralizing responses has been confirmed with two different assays without any overall significant difference between the Comirnaty and the SΔRBD group up to four weeks pe.

## Discussion

Current licensed vaccines and vaccine candidates tested in preclinical settings focus on either the complete S glycoprotein or RBD^48^. Notably, RBD is highly immunodominant and targeted by approximately 90% of the neutralizing antibody response present in SARS-CoV-2 immune sera post-infection^13,16,17^ or generated upon vaccination^18^. However, continuous SARS-CoV-2 evolution has led to the emergence of multiple variants of concern (VOCs), including Alpha, Beta, Gamma, Delta, and Omicron^49^. Notably, Omicron has further mutated into many subvariants, including BA2 and XBB variants, which are prone to immune evasion provided by current vaccines and therapeutic antibodies targeting the original strain or previous VOCs^27,50–54^. As a result, the vaccine- or earlier virus infection-acquired antibody repertoire is likely insufficient to fully protect against later VOCs while remaining largely efficient to protect against severe covid-19 disease^55^.

In order to focus the immune response away from RBD, which accounts for 44% of S mutations (15 in total) in the original Omicron variant B.1.1.529, we developed a SΔRBD antigen coupled to lipid vesicles as a nanoparticle vaccine candidate. We show that the structure of S without RBD folds into the prefusion S conformation^46^, which was stabilized by crosslinking, revealing one major site that crosslinks inter S2 subunits as reported for complete S^56^.

Since a large number of the population has been vaccinated at least twice^57^, we evaluated if a single booster dose with SΔRBD VLPs enhances S2-specific antibodies and analyzed their contribution to neutralization after two mRNA vaccinations with Comirnaty. Intermuscular delivery of the vaccine-induced robust immunogenicity, mobilizing both humoral and cellular compartments. Throughout the study, no adverse events were recorded in the animals, supporting the safety of the vaccine. Total antibody titers were similar in both the Comirnaty and the SΔRBD groups, indicating that the second boost with SΔRBD VLPs is immunogenic and boosts non-RBD antibodies targeting S2 and likely NTD to similar overall S-antibody titers in both groups. NTD is highly glycosylated, and the early Omicron strain BA1.1.529 contains 8 mutations compared to 15 in RBD and two major neutralizing sites, including antigenic supersite 1 that is recognized by most NTD-specific neutralizing antibodies^58–61^. Although, we have not distinguished between NTD and S2 antibodies in this study, S2 has been reported to be immunogenic harboring neutralizing antibody epitopes^41,62^ including stem-helix recognizing broadly neutralizing antibodies^42,63–65^ and fusion peptide targeting antibodies^66^. Notably, the analysis of 14 million SARS Cov2 spike protein sequences identified 17 conserved epitopes, of which 11 are localized on S2. Some of them are partly shielded by glycans, and removal of the stem glycans improved broad neutralization upon mRNA vaccination, indicating that highly conserved S2 epitopes are attractive targets for coronavirus vaccine development^67^. In line with these data, immunization of mice with stabilized S2 antigens in prime-boost schemes conferred protection from mortality upon infection using double boosts with S2 antigens. Notably deglycosylation of S2 antigens induced weak cross neutralization of MERS CoV^68^. Further studies showed that S2 heptad repeat antigens induce broadly neutralizing antibodies^69^ and S2 DNA vaccination of mice previously vaccinated with SARS-CoV-2 Wuhan-based S induced a broader neutralizing antibody response than a Wuhan S vaccination boost^70^.

We show that serum neutralization activities in the Comirnaty and the SΔRBD groups were similar, suggesting indirectly that non-RBD antibodies generated by the SΔRBD VLP boost contribute to neutralization of wild type and omicron strains IN2, BA.1, and BA.4/5. In line with neutralization results, reduced viral burden was measured in nasal, tracheal, and lung fluids after challenge with omicron variant XBB3, a subvariant of BA.2.75, which is also closely related to BA.4/5. Notably, XBB subvariants have been reported to efficiently evade bivalent mRNA booster as well as previous mRNA vaccination (Wuhan strain) followed by a breakthrough Omicron infection^27,38,39,71^. Furthermore, the SΔRBD group revealed gRNA and sgRNA levels in BAL supernatants below our quantification capacities, which may indicate that the SΔRBD VLP boost can protect from lung infection. Although the number of animals per group is low, we suggest that the observed trend of the study indicates an important difference between the Comirnaty and SΔRBD groups because lung infection is a hallmark of Covid-19. Although most omicron variants show reduced lung infection associated with virus attenuation, others can revert, such as BA2.86, revealing efficient lung cell infection^72,73^. Vaccine-induced protection from lung infection may be due to the liposomal vaccine formulation that can induce mucosal immune responses upon systemic immunization^74^. In agreement with this hypothesis, we detected in a previous study modest IgG and IgA titers in tracheal fluids upon immunization with VLPs harboring full-length S, which provided sterilizing immunity upon virus challenge^56^. Because immunity was not sterilized in the present study, IgA abundance and specificity were not investigated. Currently, licensed SARS-CoV-2 vaccines do not induce mucosal immunity and thus are poorly efficient at preventing infection or airway shedding of the virus, although different approaches suggest the feasibility of mucosal protection in preclinical models with life-attenuated vaccines^75–77^ and exosome-based RBD-decorated antigens^78^.

In summary, we show that SΔRBD VLPs are highly immunogenic and effectively redirect the immune response away from immunodominant RBD. Our results hint at a skewed repertoire towards non-RBD epitopes. Although repertoire reorientation remains minimal in this study, future refinement, including several boosts using the SΔRBD vaccine, different formulations, routes of immunization, or vaccination schedules, could lead to better diversification. If proven successful, this strategy, in combination with existing vaccines, could lead to a lower threshold of immune escape from the SARS-CoV-2 virus. Furthermore, a major drawback of currently licensed vaccines is that they are subject to cellular and humoral immunity waning over time^79,80^. Therefore, mRNA priming followed by boosting with heterologous vaccines, such as protein-based ones, might offer several advantages, such as broader, more robust, and longer-lasting immune responses^81–83^.

## Methods

### Cell lines

HEK293T (ATCC CRL-11268)^84^ and HEK293F (Thermo Fisher Scientific) and are human embryonic kidney cell lines. HEK293F cells are adapted to grow in suspension. HEK293F cells were cultured at 37 °C with 8% CO_2_ and shaking at 125 rpm in 293FreeStyle expression medium (Life Technologies). HEK293T cells were cultured at 37 °C with 5% CO_2_ in flasks with DMEM supplemented with 10% fetal bovine serum (FBS), streptomycin (100 μg/mL) and penicillin (100 U/mL). HEK293T/ACE2 cells^85^ are a human embryonic kidney cell line expressing human angiotensin-converting enzyme 2. HEK293T/ACE2 cells were cultured at 37 °C with 5% CO_2_ in flasks with DMEM supplemented with 10% FBS, streptomycin (100 μg/mL) and penicillin (100 U/mL). VeroE6 cells (ATCC CRL-1586) are kidney epithelial cells from African green monkeys. VeroE6 cells were cultured at 37 °C with 5% CO_2_ in DMEM supplemented with or without streptomycin (100 μg/mL) and penicillin (100 U/mL) and with or without 5 or 10% FBS, and with or without TPCK-trypsin. PBMC were isolated from macaque sera and cultured in RPMI1640 Glutamax+ medium (Gibco) supplemented with 10% FBS.

### Viruses

SARS-CoV-2 virus (XBB.3, a BA2.75 subvariant) was isolated by the National Reference Center for Respiratory Viruses (Institut Pasteur, Paris, France) as previously described^86^ and produced by two passages on Vero E6 cells in DMEM (Dulbecco’s Modified Eagles Medium) without FBS, supplemented with 1% P/S (penicillin at 10,000 U mL^-1^ and streptomycin at 10,000 μg mL^-1^) and 1 μg mL^-1^ TPCK-trypsin at 37 °C in a humidified CO_2_ incubator and titrated on Vero E6 cells. Whole genome sequencing was performed as described^86^ with no modifications observed compared with the initial specimen and sequences were deposited after assembly on the GISAID EpiCoV platform under accession number ID EPI_ISL_410720. The SARS-CoV-2 hCoV-19/France/HDF-IPP53307/2022, Omicron, lineage XBB.3 is now available at https://www.european-virus-archive.com under Ref-SKU: 017V-04974.

### Ethics and biosafety statement

Cynomolgus macaques (Macaca fascicularis) originating from Mauritian AAALAC-certified breeding centers were used in this study (Supplementary Table 2). All animals were housed in IDMIT infrastructure facilities (CEA, Fontenay-aux-roses), under BSL-2 and BSL-3 containment when necessary (Animal facility authorization #D92-032-02, Préfecture des Hauts de Seine, France) and in compliance with European Directive 2010/63/EU, the French regulations and the Standards for Human Care and Use of Laboratory Animals, of the Office for Laboratory Animal Welfare (OLAW, assurance number #A5826-01, US). The protocols were approved by the institutional ethical committee “Comité d’Ethique en Expérimentation Animale du Commissariat à l’Energie Atomique et aux Energies Alternatives” (CEtEA #44) under statement number A20_061. The study was authorized by the “Research, Innovation, and Education Ministry” under registration number APAFIS#28946-2021011312169043 v2.

### Animals and study design

Cynomolgus macaques were randomly assigned to three experimental groups. One Comirnaty vaccinated group (*n* = 3) received three doses of the Pfizer/BioNTech Comirnaty (30 µg, Comirnaty group) and the second group (*n* = 3) received two doses of Comirnaty and a third dose of 50 µg of SΔRBD-LVs adjuvanted with 250 µg of MPLA liposomes (Polymun Scientific, Klosterneuburg, Austria) diluted in PBS at weeks 0, 4 and 11, while control animals (*n* = 4) two received only PBS and two nothing. Vaccinated animals were sampled in blood at weeks -2,0, 2, 4, 6, 11, 13, 15, 19, 21 and 23. At week 24, all animals were exposed to a total dose of 10^5^ pfu of SARS-CoV-2 virus (XBB.3 strain) via the combination of intranasal and intra-tracheal routes (0,25 mL in each nostril and 4,5 mL in the trachea, i.e., a total of 5 mL; day 0), using atropine (0.04 mg/kg) for pre-medication and ketamine (5 mg/kg) with medetomidine (0.042 mg/kg) for anesthesia. Nasopharyngeal and tracheal swabs were collected at days 1, 2, 3, 4, 7, 10, 14, and 29 days past exposure (dpe) while blood was taken at days 2, 4, 7, 10, 14, and 29 dpe. Bronchoalveolar lavages (BAL) were performed using 50 mL sterile saline on 3 dpe. Blood cell counts, hemoglobin, and hematocrit, were determined from EDTA blood using a DHX800 analyzer (Beckman Coulter).

For all handling procedures, animals were anesthetized using ketamine hydrochloride (Imalgen® 1000 5 mg/kg) associated with medetomidine hydrochloride (Domitor® 0.05 mg/kg) by intra-muscular route. Just after the end of the manipulations, atipamezole hydrochloride (Antisedan® 0.05 mg/kg) was administered to induce recovery from anesthesia. Animals were euthanized at day 28 post SARS-CoV-2 exposure by an intravenous overdose of pentobarbital (Dolethal® 180 mg/kg) into the saphenous vein.

### Protein expression and purification

The SARS-CoV-2 S gene (Wuhan-Hu-1 Spike) encoding residues 1-331-GSGSGS-530-1208 (RBD residues 332-529 deleted) with proline substitutions at residues K986 P, V987P, F817P, A892P, A899P, A942P (“6P”), a “GSAS” substitution at the furin cleavage site (residues 682-685) a C-terminal T4 fibritin trimerization motif and a linker GSGHHHHHHHHGSGC containing 8xHis-tag^4,46^ was transiently expressed in FreeStyle293F cells (Thermo Fisher Scientific) using polyethylenimine (PEI) 1 μg/μL for transfection. Supernatants were harvested five days post-transfection, centrifuged for 30 min at 5000 g, and filtered using 0.45 μm filters (ClearLine®). SARS-CoV-2 S protein was purified from the supernatant by Ni^2+^-Sepharose chromatography (Excel purification resin, Cytiva) in buffer A (50 mM HEPES pH 7.4, 200 mM NaCl) and eluted in buffer B (50 mM HEPES pH 7.4, 200 mM NaCl, 500 mM imidazole). Eluted SARS-CoV-2 S containing fractions were concentrated using Amicon Ultra (cut-off: 30 KDa) (Millipore) and further purified by size-exclusion chromatography (SEC) on a Superose 6 column (GE Healthcare) in buffer A or in PBS. The SARS-CoV-2 S RBD domain (residues 319 to 541) was expressed in EXPI293 cells by transient transfection according to the manufacturer’s protocol (Thermo Fisher Scientific) and as described^56^. Briefly, supernatants were harvested five days after transfection and cleared by centrifugation. The supernatant was passed through a 0.45 μm filter, and RBD was purified using Ni^2+^-chromatography (HisTrap HP column, GE Healthcare) in buffer C (20 mM Tris pH 7.5 and 150 mM NaCl buffer) followed by a washing step with buffer D (20 mM Tris pH 7.5 and 150 mM NaCl buffer, 75 mM imidazole) and elution with buffer E (20 mM Tris pH 7.5 and 150 mM NaCl buffer, 500 mM imidazole). Eluted RBD was further purified by SEC on a Superdex 75 column (GE Healthcare) in buffer C. Protein concentrations were determined using an absorption coefficient (A1%,1 cm) at 280 nm of 10.4 and 13.06 for S protein and RBD, respectively, using ProtParam (https://web.expasy.org/).

### SARS-CoV-2 SΔRBD crosslinking

SΔRBD protein at 1 mg/mL in PBS was cross-linked with 4% formaldehyde (FA) (Sigma) overnight at room temperature. The reaction was stopped with 1 M Tris HCl pH 7.4 adjusting the sample buffer to 7.5 mM Tris/HCl pH 7.4. FA was removed by PBS buffer exchange using 30 KDa cut-off concentrators (Amicon). FA crosslinking was confirmed by separating SARS-CoV-2 FA- SΔRBD on a 10% SDS-PAGE under reducing conditions.

### SΔRBD protein coupling to liposomes

Liposomes for conjugating S protein were prepared as described previously^56^. Briefly, liposomes were composed of 60% of L-α-phosphatidylcholine, 4% His tag-conjugated lipid, DGS-NTA-(Ni^2+^), and 36% cholesterol (Avanti Polar Lipids). Lipid components were dissolved in chloroform, mixed, and placed for 2 h in a desiccator under vacuum at room temperature to obtain a lipid film. The film was hydrated in filtered (0.22 µm) PBS, and liposomes were prepared by extrusion using membrane filters with a pore size of 0.1 μm (Whatman Nuclepore Track-Etch membranes). The integrity and size of the liposomes were analyzed by negative staining-EM. For protein coupling, the liposomes were incubated overnight with FA-S or S protein in a 3:1 ratio (w/w). Free FA-S protein was separated from the FA-S-proteoliposomes (SΔRBD-LVs) by sucrose gradient (5-40%) centrifugation with a SW55 rotor at 40,000 rpm for 2 h. The amount of protein conjugated to the liposomes was determined by Bradford assay and SDS-PAGE densitometry analysis comparing SΔRBD-LV bands with standard S protein concentrations.

### S protein thermostability

Thermal denaturation of SARS-CoV-2 S ‘6 P’, SΔRBD, and FA-SΔRBD was analyzed by differential scanning fluorimetry (DSF) coupled to back scattering using a Prometheus NT48 instrument (Nanotemper Technologies, Munich, DE). Protein samples were first extensively dialyzed against PBS pH 7.4, and the protein concentration was adjusted to 0.3 mg/mL. 10 μL of the sample were loaded into the capillary, and intrinsic fluorescence was measured at a ramp rate of 1 °C/min with an excitation power of 30%. Protein unfolding was monitored by the changes in fluorescence emission at 350 and 330 nm. The thermal unfolding midpoint (Tm) of the proteins was determined using the Prometheus NT software.

### Negative stain electron microscopy

Protein samples were visualized by negative-stain electron microscopy (EM) using 3–4 µL aliquots containing 0.1–0.2 mg/mL of protein. Samples were applied for 10 s onto a mica carbon film and transferred to 400-mesh Cu grids that had been glow discharged at 20 mA for 30 s and then negatively stained with 2% (wt/vol) Uranyl Acetate (UAc) for 30 s. Data were collected on a FEI Tecnai T12 LaB6-EM operating at 120 kV accelerating voltage at 23k magnification (pixel size of 2.8 Å) using a Gatan Orius 1000 CCD Camera. Preliminary 2-D class averaging was performed with Relion^87^ using 9800 particles, a pixel size of 3.9 Å/pixel, and a box size of 200 pixels.

### Cryo-electron microscopy

#### Data collection

3.5 µL of SΔRBD sample were applied to 1.2/1.3 C-Flat (Protochips Inc) holey carbon grids or 1.2/1.3 Ultrafoil grids (Quantifoil MicroTools GmbH, Germany) and plunged frozen in liquid ethane with a Vitrobot Mark IV (Thermo Fisher Scientific) (6 s blot time, blot force 0). The sample was observed at the beamline CM01 of the ESRF (Grenoble, France)^88^ with a Titan Krios G3 (Thermo Fischer Scientific) at 300 kV equipped with an energy filter (Bioquantum LS/967, Gatan Inc, USA) (slit width of 20 eV). Movies were recorded automatically on two different grids with a K3 direct detector (Gatan Inc., USA) with EPU (Thermo Fischer Scientific). A total of 11051 movies were recorded for a total exposure time of 1.6 s with 40 frames per movie and a total dose of ~40 e − /Å^2^. The magnification was 105,000x (0.84 Å/pixel at the camera level). The defocus of the images varied between −1.0 and −2.5 μm.

#### 3D reconstruction

The movies were first drift-corrected with motioncor2^89^. CTF estimation was done with GCTF^90^. The remaining image processing was performed with RELION 4^91^. An initial set of particles (box size of 86 pixels, sampling of 3 Å/pixel) was obtained by auto-picking. After two rounds of 2D classification, the particles from the best-looking 2D class averages were used to generate an ab-initio 3D map (with C1 symmetry), which was then refined to 6.2 Å imposing C3 symmetry. The 3D map displays the features expected for SΔRBD, and an atomic model of S could un-ambiguously be docked in the map. The particles used to generate that first 3D map were then used to train a picking model and pick all the micrographs with Topaz^92^. Approximately 1.1 M of particles were extracted, and a first 3D classification (C1 symmetry, 5 classes, and a circular mask diameter of 210 Å) allowed the identification of a subset of 200,114 particles, which were then re-extracted (box size of 400 pixels, sampling of 0.84 Å/pixel). A 3D refinement (C3 symmetry) gave a 3D reconstruction at 4 Å resolution. Refinement of CTF parameters, particle polishing, and a second round of CTF parameter’s refinement further improved the resolution to 3.1 Å. A final 3D classification (C1 symmetry, 3 classes, and a circular mask diameter of 210 Å) allowed the identification of a subset of 87,324 particles which then yielded the final 3D reconstruction at 3.0 Å resolution. The resolution was determined by Fourier Shell Correlation (FSC) at 0.143 between two independent 3D maps. The local resolution was calculated with blocres^93^ and found to be between 2.8 and 4.4 Å. The final 3D map was sharpened with DeepEMhancer for display purpose^94^.

#### Model refinement

The atomic model of the S protein, paraformaldehyde fixed, in the closed conformation (PDB 7Q1Z)^56^ was rigid-body fitted inside the cryo-EM density map in CHIMERA^95^. The residues corresponding to the RBD domains were deleted. The atomic coordinates were then refined with Rosetta^96^ and PHENIX^97^. The refined atomic models were visually checked and adjusted (if necessary) in COOT^98^. The final model was validated with MOLPROBITY^99^. The data collection and atomic model statistics are summarized in Supplementary Table 1.

### Virus quantification in NHP samples

Upper respiratory (nasopharyngeal and tracheal) specimens were collected with swabs (Viral Transport Medium, CDC, DSR-052-01). Tracheal swabs were performed by insertion of the swab above the tip of the epiglottis into the upper trachea at approximately 1.5 cm of the epiglottis. All specimens were stored at −80 °C until extraction and analysis. Extraction was done using the NucleoSpin™ Virus Core Kit (Macherey-Nagel, Dueren, Germany) according to the manufacturer’s instructions. Samples were then analyzed by RT-qPCR with a virus standard concentration range containing an RdRp gene fragment, including the RdRp-IP4 RT-PCR target sequence IP4-SARS-CoV-2-R CTG GTC AAG GTT AAT ATA GG, IP4-SARS-CoV-2--F: GGT AAC TGG TAT GAT TTC G and SARS6CoV-2 IP4 probe: FAM-TCA TAC AAA CCA CGC CAG G-BHQ1. SARS-CoV-2 E gene subgenomic mRNA (sgRNA) levels were assessed by RT-qPCR using primers and probes previously described^100,101^: leader-specific primer sgLeadSARSCoV2-F CGATCTCTTGTAGATCTGTTCTC, E-Sarbeco-R primer ATATTGCA GCAGTACGCACACA and E-Sarbeco probe HEX-ACACTAGCCATCCTTACTGCGCTTCG-BHQ1. The protocol describing this procedure for the detection of SARS-CoV-2 is available on the WHO website (https://www.who.int/docs/default-source/coronaviruse/whoinhouseassays.pdf). RT-qPCR was performed using the SuperScript® III Platinium® One-step Quantitative RT-qPCR System kit (Life Technologies, CA, USA) and C1000 Thermal Cylcer with CF96 optic module (Bio-Rad Laboratores, Diamed GmbH, Switzerland) according to the manufacturer’s instruction. Data were then analyzed using CFX Maestro (v2.2, Bio-Rad).

### ELISA

Serum antibody titers specific for soluble WT S glycoprotein, SΔRBD, and for RBD were determined using an enzyme-linked immunosorbent assay (ELISA). Briefly, 96-well microtiter plates (F96 Maxisorp NUNC Immunoplate #442404) were coated with 50 µL at 1 µg/mL of WT S, SΔRBD or RBD proteins at 4 °C overnight in PBS and blocked with 3% BSA for 1 h at room temperature after 3 washes with PBS Tween-20 0.05%. Serum dilutions were added to each well, and plates were washed 5 times with PBS Tween. An alkaline phosphatase-conjugated anti-monkey IgG (Sigma Aldrich, # A1929) was then diluted 1/10,000 and incubated for 1 h, before excess Ab was washed out and PNPP substrate was added (Interchim, # UP 664791). Absorbance was read at 1 h15, at 405 nm. Antibody titers were expressed as ED50 (Effective Dilution 50-values) and were determined as the serum dilution at which IgG binding was reduced by 50%. ED50 was calculated from crude data (O.D) after normalization using GraphPad Prism (version 9) “log(inhibitor) vs normalized response” function.

### Pseudovirus neutralization assay

Neutralization activity was tested using a pseudovirus neutralization assay, as previously described^84^. Shortly, pseudoviruses were produced by co-transfecting the pCR3 SARS-CoV-2-SΔ19 (Wuhan-Hu-1, GenBank MN908947.3 with amino acid substitution D614G) expression plasmid with the pHIV-1_NL43_ ΔEnv-NanoLuc reporter virus plasmid in HEK293T cells (ATCC, CRL-11268), as previously described^85^. Cell supernatant containing the pseudovirus was harvested 48 h post transfection and stored at −80 °C until further use. For the neutralization assay itself, HEK293T/ACE2 cells^85^ were seeded at a density of 20,000 cells/well in a 96-well plate coated with 50 μg/mL poly-L-lysine one day prior to the start of the neutralization assay. Serum was serially diluted in cell culture medium (DMEM (Gibco), supplemented with 10% FBS, penicillin (100 U/mL), streptomycin (100 μg/mL), and GlutaMax (Gibco)), subsequently mixed in a 1:1 ratio with pseudovirus and incubated for 1 h at 37 °C. Next, these mixtures were added to the cells in a 1:1 ratio and incubated for 48 h at 37 °C, followed by cell lysis to measure the luciferase activity in cell lysates using the Nano-Glo Luciferase Assay System (Promega) and GloMax system (Turner BioSystems). Relative luminescence units (RLU) were normalized to the positive control wells where cells were infected with pseudovirus in the absence of serum. The neutralization titers (IC_50_) were determined as the antibody concentration at which infectivity was inhibited by 50%, respectively, using a non-linear regression curve fit (GraphPad Prism software version 8.3).

### RBD-specific antibody serum depletion

3 mg of RBD was coupled to cyanogen-bromide-activated Sepharose ^TM^ 4 Fast Flow (Sigma-Aldrich) following the manufacturers’ protocol in order to generate RBD-sepharose affinity columns. 200 µL of serum was successively three times passed over 100 µL RBD-sepharose, washed with 100 µL PBS. The flow through was collected and concentrated after the third passage to 200 µL. Complete RBD depletion was confirmed by ELISA using RBD-coated plates as described above.

### Electrochemiluminescence-based neutralization assay

Neutralizing antibody titers were measured using the Meso Scale Discovery MSD V-PLEX SARS-CoV-2 Key Variant Spike Panel 1 ACE2 competition assay kit according to the manufacturer’s instructions. This technique permits to measure the activity of antibodies capable of blocking the binding of ACE2 to its cognate receptor from the Wuhan virus and from (B.1.1.7), (B.1.1.529; BA.1), (B.1.351), (B.1.617.2; AY.4) Alt Seq 2, (BA.2), (BA.2.12.1), (BA.2.75), and (BA.5) variants. Plates were blocked with MSD Blocker A and washed. The assay calibrator (COVID-19 neutralizing antibody; monoclonal antibody against S protein), control sera, and test sera samples diluted 1 in 10 or 1 in 100 in assay diluent were added to the plates. Following a 2 h incubation period, the plates were washed, and MSD SULFO-TAG™ conjugated ACE-2 protein was added. The plates were then read using a MESO® QuickPlex SQ 120MM Reader. The calibration curve was used to calculate neutralizing antibody concentrations in samples by backfitting the measured electrochemiluminescence (ECL) signals to the calibration curve. Data analysis was conducted using the Discovery Workbench software (v4.0 MSD, Rockville, USA), and the results were expressed as units (U)/mL.

### Antigen-specific IFNγ producing T cell assays using ELISpot

Monkey IFN-γ ELISpot PRO kit (Mabtech Monkey IFN-γ ELISpot pro, #3421M-2APT) was used following the manufacturer’s instructions. 2 ×10^5^ freshly isolated PBMCs were added to each well. Cells were stimulated using SARS-CoV-2 peptide pools in the culture medium at a final dilution of 2 μg/mL. Before the challenge, 2 pools of 158 and 157 peptides derived from a peptide scan through the Spike glycoprotein of SARS-CoV-2 were used. After the challenge, an additional pool of 102 peptides derived from a peptide scan through Nucleoprotein was used. PMA/ionomycine (62,5 ng/mL and 714,29 ng/mL respectively) was used as positive control and complete RPMI medium (RPMI1640 Glutamax+, Gibco; supplemented with 10% FBS) alone as a negative control. Plates were incubated for 18 h at 37 °C in an atmosphere containing 5% CO2 and then washed 5 times with PBS. Alkaline phosphatase linked anti-IFN-γ antibody was added and the plates were incubated 2 h at 37 °C. Plates were washed 5 times with PBS, and spots were developed by adding 0.45 μm-filtered ready-to-use BCIP/NBT-plus substrate solution. The spots were counted with an Automated IRIS™ FluoroSpot/ELISpot Reader (Mabtech AB, Stockholm, Sweden) at CEA.

### Antigen-specific T-cell assays using flow cytometry

To analyze the SARS-CoV-2 protein-specific T cell, 15-mer peptides (*n* = 157 and *n* = 158) overlapping by 11 amino acids (aa) and covering the SARS-CoV-2 Spike sequence (aa 1 to 1273) were synthesized by JPT Peptide Technologies (Berlin, Germany) and used at a final concentration of 2 µg/mL.

T-cell responses were characterized by measurement the frequency of CD4 + T cells expressing IL-2 (PerCP5.5, MQ1-17H12, BD), IL-17a (Alexa700, N49-653, BD), IFN-γ (V450, B27, BD), TNF-α (BV605, Mab11, BioLegend), IL-13 (BV711, JES10-5A2, BD), CD137 (APC, 4B4, BD), CD69 (PE-, FN-50, BioLegend) and CD154 (FITC, TRAP1, BD) upon stimulation with the two peptide pools. CD3 (APC-Cy7, SP34-2, BD), CD4 (BV510, L200, BD), and CD8 (PE-Vio770, BW135/80, Miltenyi Biotec) antibodies were used as lineage markers. One million PBMC were cultured in a complete RPMI medium, supplemented with co-stimulatory antibodies (FastImmune CD28/CD49d, Becton Dickinson). The cells were stimulated with S sequence overlapping peptide pools at a final concentration of 2 μg/mL. Brefeldin A was added to each well at a final concentration of 10 µg/mL, and the plate was incubated at 37 °C, 5% CO2 for 18 h. Next, cells were washed, stained with a viability dye (LIVE/DEAD fixable Blue dead cell stain kit, ThermoFisher), and then fixed and permeabilized with the BD Cytofix/Cytoperm reagent. Permeabilized cell samples were stored at −80 °C before the staining procedure. Antibody staining was performed in a single step following permeabilization. After 30 min of incubation at 4 °C, in the dark, cells were washed in BD Perm/Wash buffer and then acquired on the LSRII cytometer (Beckton Dickinson). Analyses were performed with the FlowJo v.10 software. Data are presented a minus background where non-stimulated cells signals was subtracted to each specific signal. No responses were detected with antibodies specific for IL17a, IL13, CD137, and CD154, and hence no data was included in the analyses.

### Statistical analysis and figure preparation

Statistical significance between groups was performed using Graphpad Prism (v9.2.0). Statistical analysis of NHP gRNA, sgRNA and Ab production was carried out using Kruskal–Wallis and Mann–Whitney unpaired *t*-test in GraphPad Prism software (v9.2.0).

Figure 1 and Supplementary Fig. 2 were prepared with CHIMERA and CHIMERAX^95,102^. Figure 2a was generated with BioRender.

## Supplementary information

Supplementary information

## Data Availability

Data is provided within the manuscript (p 16) and supplementary information file, supplementary table 1: p16: The atomic coordinates and the cryo-EM map have been deposited in the Protein Data Bank (https://www.rcsb.org/) and in the Electron Microscopy Data Bank (https://www.ebi.ac.uk/emdb/) under the accession codes 8R87 and EMD-18997, respectively.

## Code availability

Data is provided within the manuscript (p 16) and supplementary information file, supplementary table 1: p16: The atomic coordinates and the cryo-EM map have been deposited in the Protein Data Bank (https://www.rcsb.org/) and in the Electron Microscopy Data Bank (https://www.ebi.ac.uk/emdb/) under the accession codes 8R87 and EMD-18997, respectively.
