# Supplementary material for: RBD-depleted SARS-CoV-2 spike generates protective immunity in cynomolgus macaques
Source: NPJ Vaccines. 2025 Mar 30;10:63. doi: 10.1038/s41541-025-01113-0 (PMC11955555; doi:10.1038/s41541-025-01113-0)
Supplement: Supplementary file 1 — Supplementary information [file 41541_2025_1113_MOESM1_ESM.pdf]

Supplementary Information

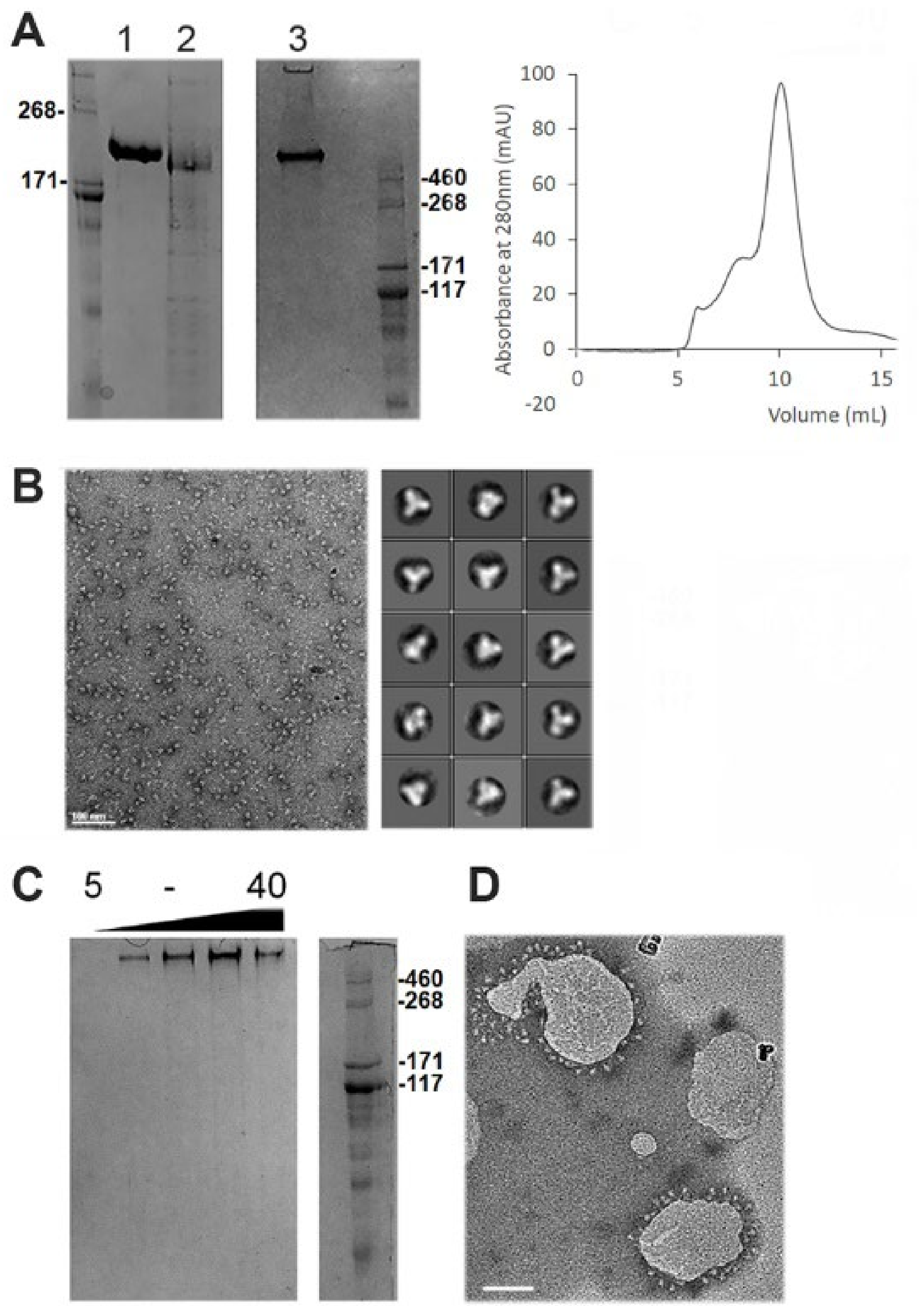

Supplementary Figure 1. Expression and characterization of the SARS_Cov-2 S glycoprotein.

(A) SDS-PAGE of purified S '6P' (lane 1), SΔRBD (lane 2) and FA cross-linked SΔRBD (lane 3). Molecular weight markers are indicated (left panels). Size exclusion chromatography (SEC) profile of SΔRBD purified on a superdex 200 column. The main peak at ~10 ml corresponds to trimeric SΔRBD (right panel).

(B) Negative staining electron microscopy of the SΔRBD (left panel) and 2-D class averages of the most populated classes (right panel) (Scale bar, 100 nm).

(C) FA-cross-linked S glycoprotein was incubated with liposomes containing 4% DGS-NTA lipids, purified by sucrose gradient density centrifugation (from 5 to 40 % sucrose) and analyzed by SDS-PAGE analysis.

(D) Negative staining electron microscopy shows decoration of the liposomes with SΔRBD; SΔRBD LVs had approximate diameters of 100 to 200 nm (scale bar, 50 nm).

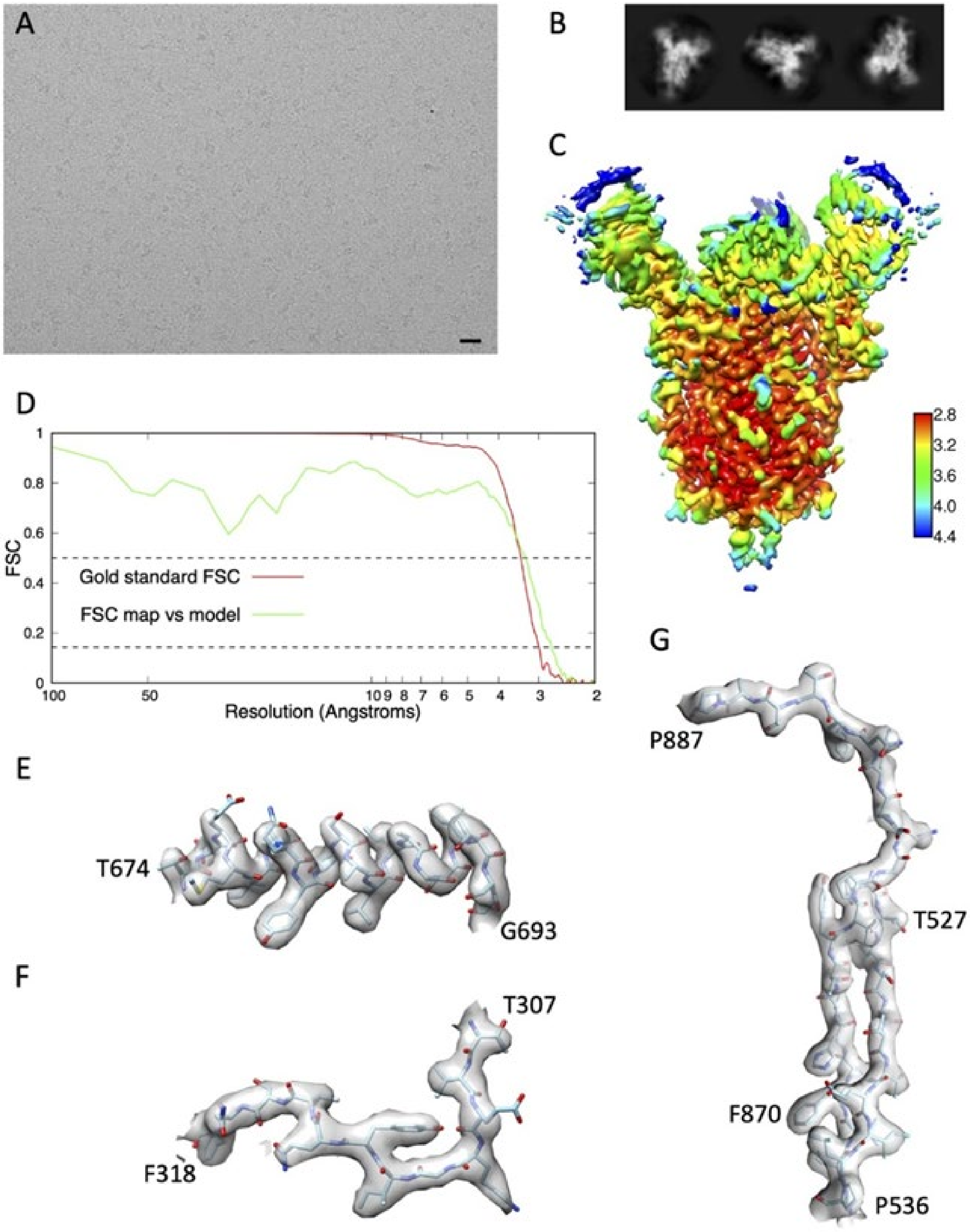

Supplementary Figure 2. Cryo-EM structure validation.
(A) Cryo-EM image of the SΔRBD trimer (scale bar 20 nm) and **(B)** 2-D class average representatives.
(C) Isosurface representation of the 3D reconstruction of the SΔRBD trimer obtained by cryo-EM colored by local resolution (color code in Å).
(D) Fourier Shell Correlation (FSC) curves calculated between two independent 3D reconstructions of the SΔRBD trimer (gold standard FSC, resolution of 3.0 Å at FSC=0.143) and between the cryo-EM 3D reconstruction and the refined atomic model (FSC map vs model, resolution of 3.3 Å at FSC=0.5).
(E, F, G) Three different zones of the SΔRBD trimer are shown to illustrate both the high resolution of the 3D reconstruction (grey isosurface) and the quality of the refined atomic model. Some residue numbers are indicated for orientation.

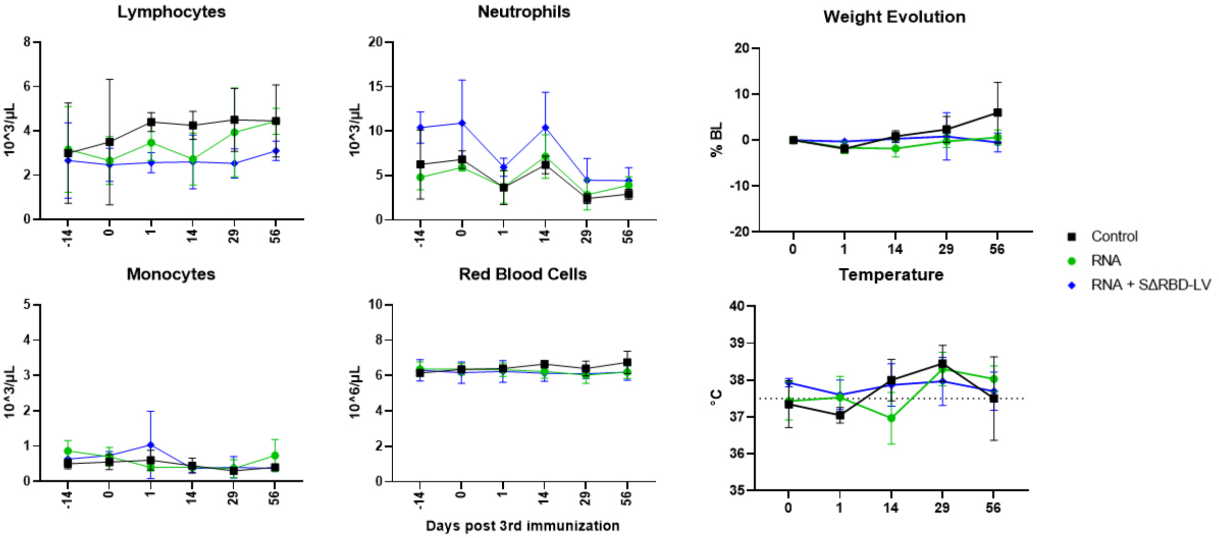

Supplementary Figure 3. Safety profile of the SΔRBD-LV vaccine.
Several clinical parameters were recorded after the different immunizations. Circulating lymphocytes, monocytes, neutrophils and Red blood cells were counted on EDTA-K3 tubes. Weight evolution was calculated as a % evolution of the considered baseline, here 14 days before the third immunization. Rectal temperature was obtained on anesthetized animals.

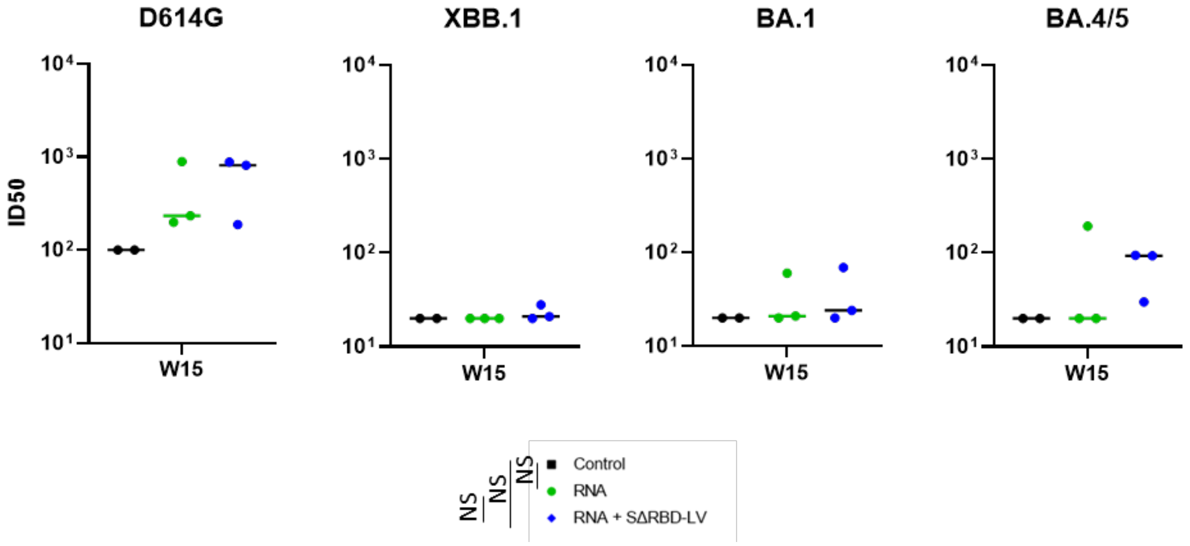

Supplementary Figure 4. Viral neutralization after anti RBD serum antibody depletion.
Neutralization of various SARS-CoV-2 strains are shown for sera collected at weeks 15 that were then depleted from RBD-specific Antibodies. Bars indicate mean titers for the three animals. Neutralization is expressed as inhibitory dilution at which 50% neutralization is achieved. Differences between groups were compared using the Mann-Whitney non parametric rank test. Data presented are from technical duplicates.

a

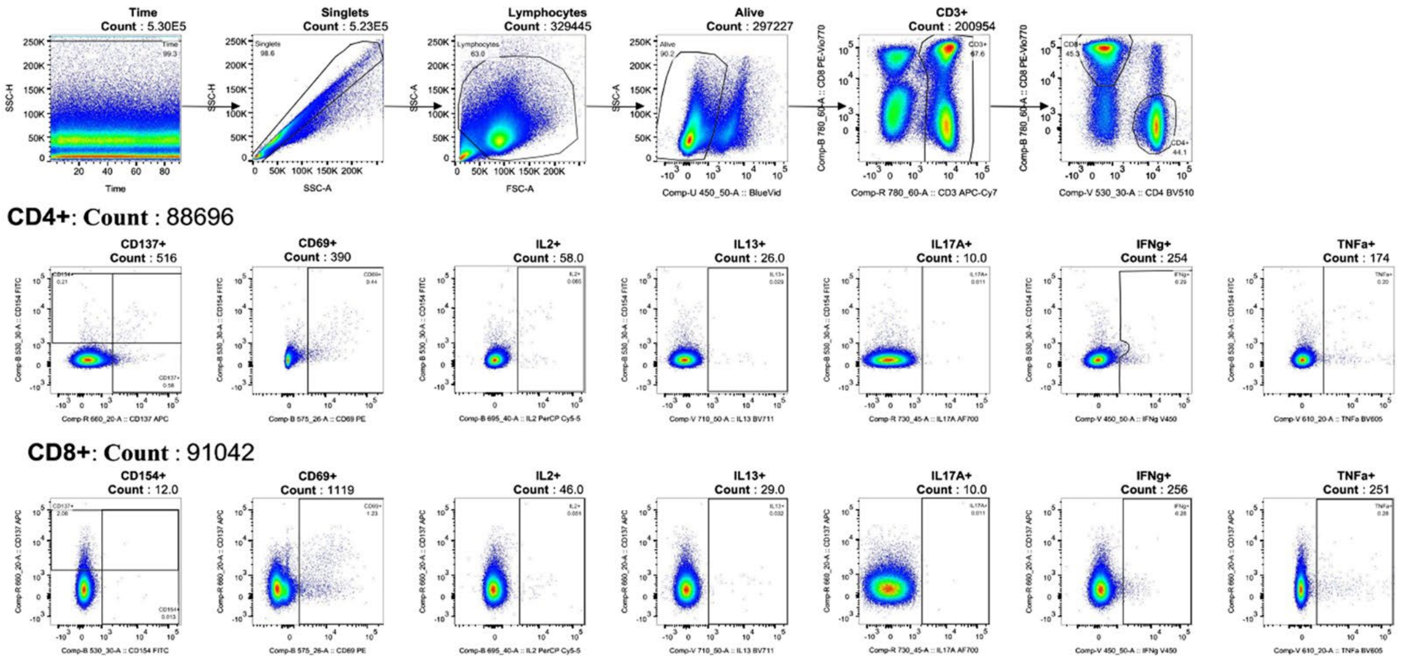
Time
Count : 5.30E5
Singlets
Count : 5.23E5
Lymphocytes
Count : 329445
Alive
Count : 297227
CD3+
Count : 200954
SSC-H
SSC-A
FSC-A
Comp-U 450_50-A :: BlueVid
Comp-R 780_60-A :: CD3 APC-Cy7
Comp-V 530_30-A :: CD4 BV510
Comp-B 780_60-A :: CD8 PE-Vio770
CD4+: Count : 88696
CD137+
Count : 516
CD69+
Count : 390
IL2+
Count : 58.0
IL13+
Count : 26.0
IL17A+
Count : 10.0
IFNg+
Count : 254
TNFa+
Count : 174
CD8+: Count : 91042
CD154+
Count : 12.0
CD69+
Count : 1119
IL2+
Count : 46.0
IL13+
Count : 29.0
IL17A+
Count : 10.0
IFNg+
Count : 256
TNFa+
Count : 251

b

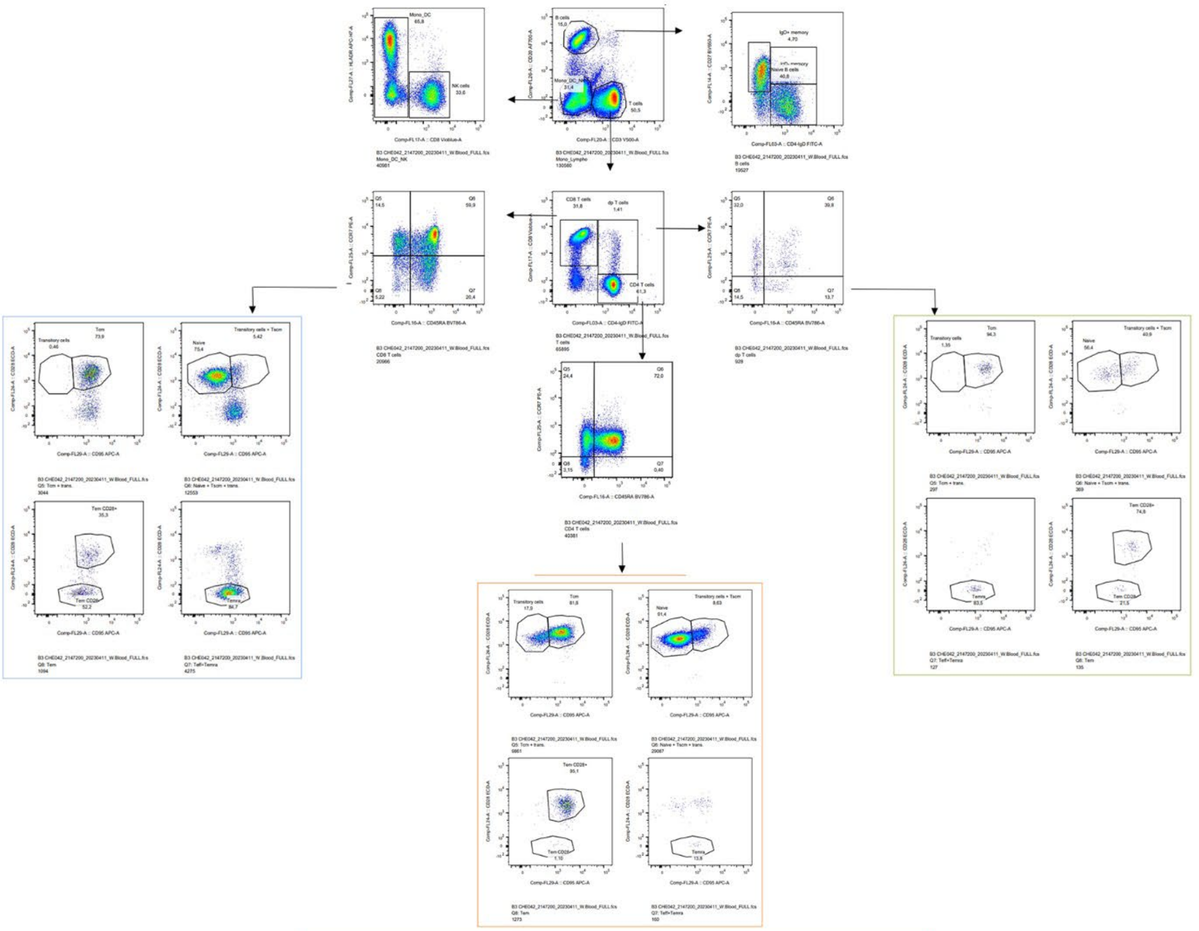

c

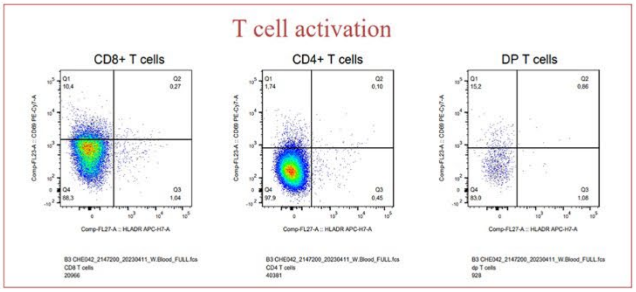
T cell activation
CD8+ T cells
CD4+ T cells
DP T cells

Supplementary Figure 5. Flow cytometry intracellular gating strategy.
(A) Stimulation-specific cytokine production by CD4+ and CD8+ T cells was characterized by flow cytometry using intra-cellular staining. Cells were gated using the following approach, with each gate defined by the result of the previous one: singlets>lymphocytes>live cells>CD3+ cells. Amongst those cells, CD4+CD8- and CD8+CD4- were gated. In each cell type, cytokine positive cells were selected, regardless of the activation status.
(B) Gating strategy of the different T cells subtypes amongst CD3+,live singlet lymphocytes. Left, CD8+ T cells, bottom, CD4+ T cells, Right CD4+CD8+ double positive (DP) T cells.
(C) Gating strategy for activated T cells, left CD8+ T cells, center, CD4+ T cells, right, DP T cells. The analysis was performed with the FlowJov 10 software.

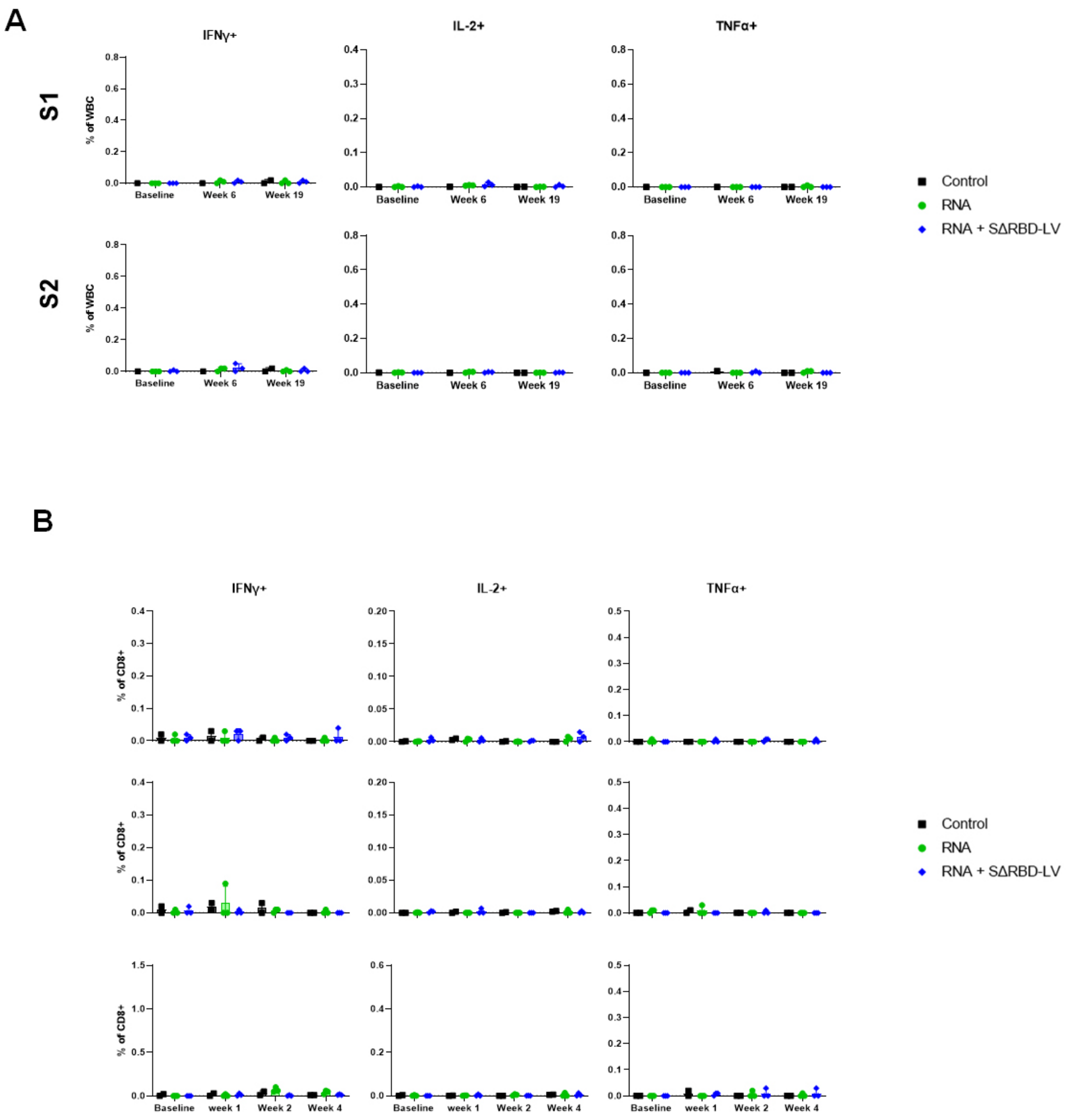

Supplementary Figure 6. CD8 T cell activation.
CD8 T cell activation was assessed at the immunization phase **(A)** and after challenge **(B)** using flow cytometry. No cytokine production was observed regardless of the stimulating peptide pool or group.

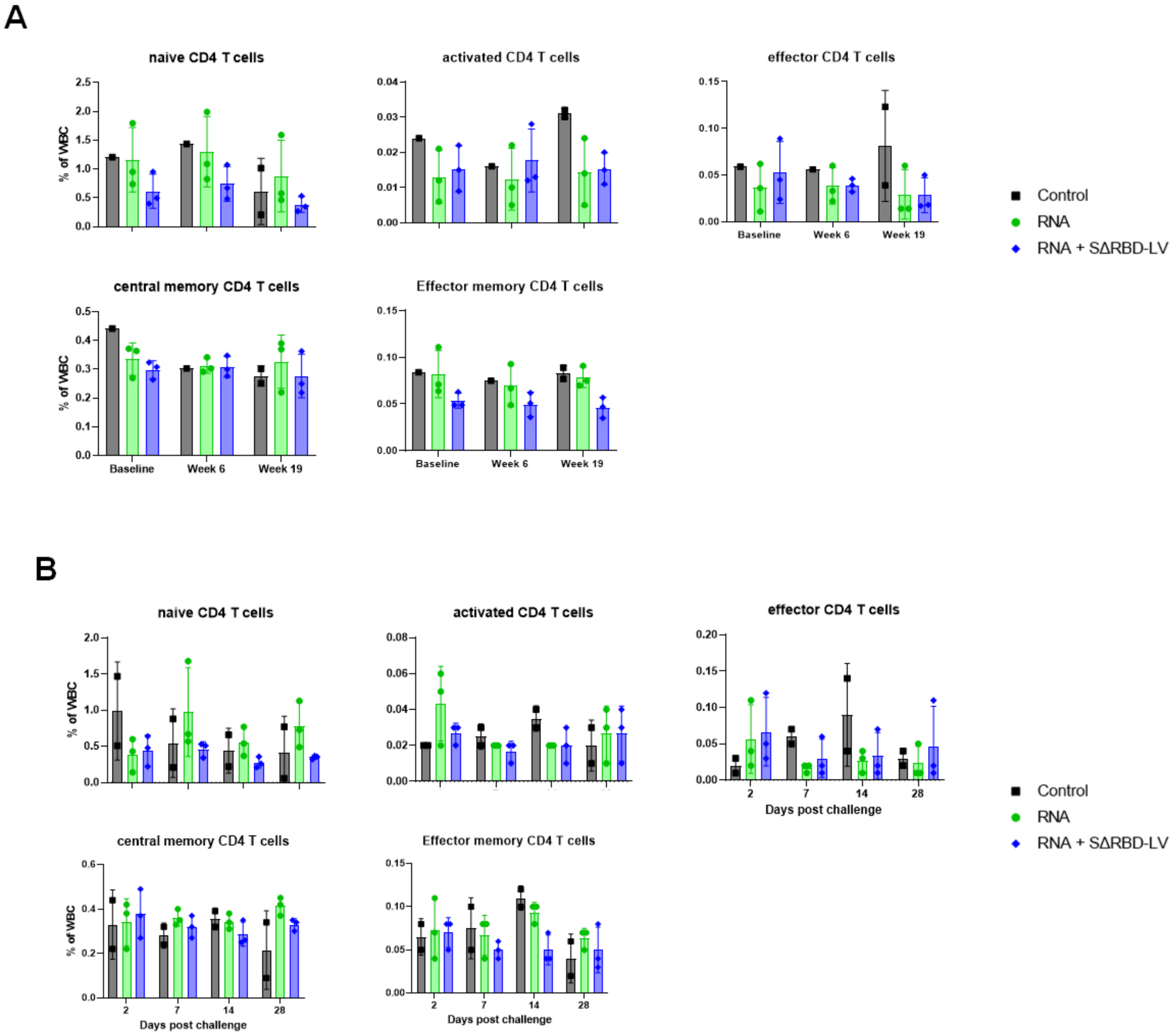

Supplementary Figure 7. CD4 T cells subtypes.

CD4 T cells subtypes were characterized using flow cytometry at the immunization phase (A) and after challenge (B). Naïve T cells were gated as CD4+CD45RA+CCR7+; central memory T cells as CD4+CCR7+CD45RA-; effector cells as CD4+CCR7-CD45RA+; effector memory as CD4+ CCR7-CD45RA-; activated T cells as CD4+HLA-DR+CD69+.

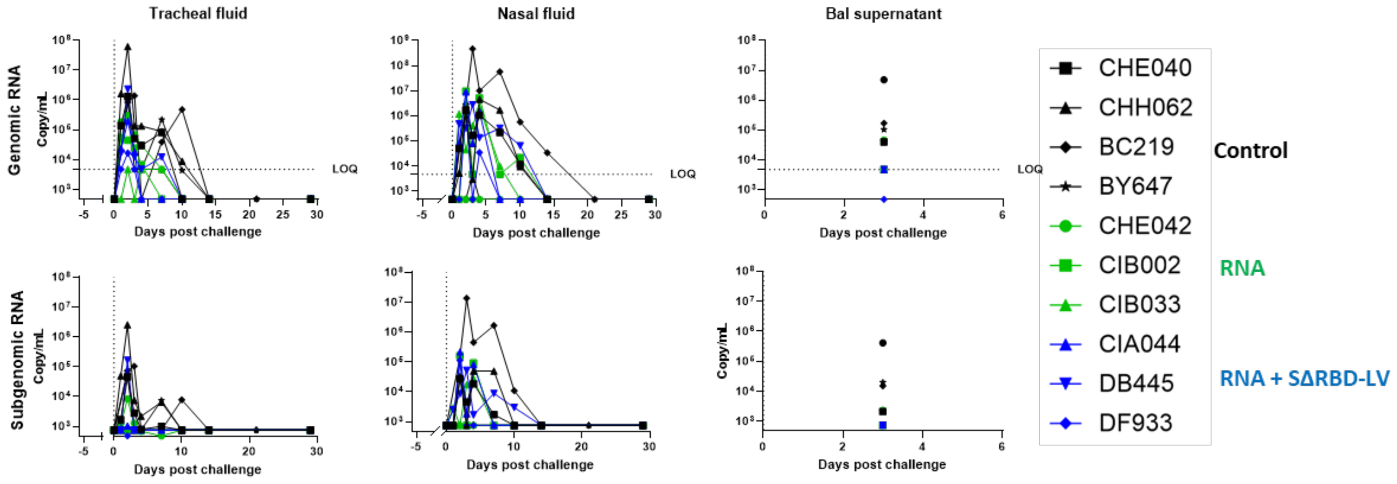

Supplementary Figure 8. Viral loads.
Nasopharyngeal, tracheal and broncho-alveolar SARS-CoV-2 viral loads from individual animals. Top row: Genomic RNA and lower row subgenomic RNA viral loads in fluids of control (black) and RNA vaccinated (green) and RNA vaccinated, SΔRBD boosted (blue) macaques after challenge. Vertical dotted lines indicate the day of immunization. The dotted line represent the lower limit of quantification (LOQ) when applicable. When data were between the LOD and the LOQ they were adjusted to the LOQ. Each animal is indicated as a different symbol.

Supplementary Table 1: Summary of data collection and atomic model statistics.

**Data collection**	
Microscope	Krios G3 (Thermo Fischer Scientific)
Voltage (kV)	300
Magnification<br>Unbinned pixel size	105,000x<br>0.84 Å/pixel
Camera	K3 (Gatan Inc)
Exposure time	1.6s
Number of frames	40
Total dose ($e^{-}/Å^{2}$)	39.7
**Image processing**	
EMDB	18997
Symmetry	C3
Final number of Particles	87,324
Map resolution in Å (FSC 0.143)	3.0
**Model statistics**	
PDB	8R87
Model to map resolution in Å (FSC 0.5)	3.3
Ramachandran favored (%)	97.9
Ramachandran outliers (%)	0.0
Rama Z score	1.19
Rotamer outliers (%)	0.14
C-beta deviations	0
Rms on bond lengths	0.0027
Rms on bond angles	0.50
Clashscore	2.4
Molprobity score	1.0

Supplementary Table 2: Description of animals, control group, Comirnaty group and SΔRBD group.

	vaccine	Gender	Date of birth	Age (years)	Weight at Day 0 post exposure (kg)	Dev. stage	Comment
CHE040	Control	F	24/05/2018	4.8	3.2	Young adult	
CHH062	Control	M	31/08/2018	4.5	5.9	Young adult	
BC219	Control	F	12/12/2006	16.1	4,89	Mature adult	Historical* control
BY647	Control	F	11/07/2010	12.5	4,84	Mature adult	Historical* control
CH042	Comirnaty	F	25/05/2018	4.8	3.5	Young adult	
CIB033	Comirnaty	F	06/02/2019	4.1	3.4	Young adult	
CIB002	Comirnaty	F	02/02/2019	4.1	3.8	Young adult	
CIA044	SΔRBD	F	22/01/2019	4.1	3.67	Young adult	
DB445	SΔRBD	F	07/09/2017	5.5	5.13	Young adult	
DF933	SΔRBD	M	19/09/2018	4.5	5.15	Young adult	

* Historical control, animals challenged in another experiment
